# Shared and Compartment‐Specific Processes in Nucleus Pulposus and Annulus Fibrosus During Intervertebral Disc Degeneration

**DOI:** 10.1002/advs.202309032

**Published:** 2024-02-25

**Authors:** Hannah Swahn, Jasmin Mertens, Merissa Olmer, Kevin Myers, Tony S. Mondala, Padmaja Natarajan, Steven R. Head, Oscar Alvarez‐Garcia, Martin K. Lotz

**Affiliations:** ^1^ Department of Molecular and Cellular Biology & Department of Molecular Medicine Scripps Research La Jolla CA 92037 USA; ^2^ Center for Computational Biology & Bioinformatics and Genomics Core Scripps Research La Jolla CA 92037 USA

**Keywords:** FOXO transcription factors, intervertebral disc (IVD), intervertebral disc degeneration (IDD), single‐cell RNA‐sequencing (scRNA‐seq), thrombospondin (THBS) signaling

## Abstract

Elucidating how cell populations promote onset and progression of intervertebral disc degeneration (IDD) has the potential to enable more precise therapeutic targeting of cells and mechanisms. Single‐cell RNA‐sequencing (scRNA‐seq) is performed on surgically separated annulus fibrosus (AF) (19,978; 26,983 cells) and nucleus pulposus (NP) (20,884; 24,489 cells) from healthy and diseased human intervertebral discs (IVD). In both tissue types, depletion of cell subsets involved in maintenance of healthy IVD is observed, specifically the immature cell subsets – fibroblast progenitors and stem cells – indicative of an impairment of normal tissue self‐renewal. Tissue‐specific changes are also identified. In NP, several fibrotic populations are increased in degenerated IVD, indicating tissue‐remodeling. In degenerated AF, a novel disease‐associated subset is identified, which expresses disease‐promoting genes. It is associated with pathogenic biological processes and the main gene regulatory networks include thrombospondin signaling and FOXO1 transcription factor. In NP and AF cells thrombospondin protein promoted expression of genes associated with TGFβ/fibrosis signaling, angiogenesis, and nervous system development. The data reveal new insights of both shared and tissue‐specific changes in specific cell populations in AF and NP during IVD degeneration. These identified mechanisms and molecules are novel and more precise targets for IDD prevention and treatment.

## Graphical abstract

Intervertebral (IVD) cells maintain tissue structure and biomechanical function while their depletion and dysfunction are key mechanisms in IVD degeneration and back pain. Using scRNA‐seq, cell type and compartment‐specific are resloved and shared mechanisms and their transcriptional regulators that are dysregulated in disease. These findings define novel genes and gene regulatory networks that enable more precise therapeutic targeting for the prevention and treatment of intervertebral disc degeneration.

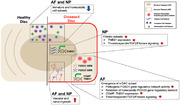

## 1 Introduction

Low back pain (LBP) is the most common cause for physical disability in adults with approximately 632 million people being affected worldwide.^[^ ^1^ ^]^ LBP is considered a serious public health burden because of its tremendous impact on socioeconomics and quality of life.^[^ ^2^ ^]^

One of the major causes that lead to chronic LBP is intervertebral disc degeneration (IDD).^[^ ^3^ ^]^ Despite research advances in LBP and IDD^[^ ^3^, ^4^ ^]^ there is currently no treatment available to prevent disease development or progression.^[^ ^5^ ^]^ Pharmacologic treatments of IDD mainly address pain and include non‐steroidal anti‐inflammatory drugs, muscle relaxants, and other pain medications, including opioids. Even though drug treatment provides effective short‐term pain relief, chronic LBP often persists in patients together with loss of function and compromised quality of life. A more promising approach is to maintain or restore cellular and extracellular matrix (ECM) homeostasis of the intervertebral disc (IVD).^[^ ^6^ ^]^

Histology and magnetic resonance imaging have shown an association between LBP and lumbar IDD.^[^ ^4^, ^7^ ^]^ The IDD process is thought to start within the nucleus pulposus (NP)^[^ ^8^ ^]^ but subsequently involves the other tissue compartments, including annulus fibrosus (AF) and endplate (EP). IDD is a complex and chronic process that begins early in adulthood caused by multiple stresses^[^ ^9^ ^]^ and involves a cascade of cellular, molecular, and transcriptomic changes.^[^ ^6^, ^10^ ^]^ Key features of IDD are ECM degradation^[^ ^11^ ^]^ and changes in the cellular environment within the IVD,^[^ ^12^ ^]^ an increased number of senescent cells,^[^ ^13^ ^]^ inflammation,^[^ ^14^ ^]^ abnormal fibrotic ECM synthesis^[^ ^15^ ^]^ and in‐growth of nociceptive nerves and blood vessels.^[^ ^16^ ^]^

While some specific molecular mediators and pathways have been correlated with functional and structural changes in the degenerated IVD, there is limited understanding of regulatory mechanisms in IDD.^[^ ^17^ ^]^ To gain a deeper insight into the transcriptional networks necessary to maintain a healthy disc and changes in expression of regulatory genes, the generation of a transcriptomic map of the IVD tissues is essential.^[^ ^18^ ^]^ Single‐cell RNA sequencing (scRNA‐seq) is a powerful tool for the comprehensive analyses of the tissue specific genetic architecture^[^ ^19^ ^]^ and to enable more precise therapeutic targeting of disease‐promoting cells and their abnormal gene regulatory mechanisms. For this study, we applied scRNA‐seq to cells isolated from surgically separated AF and NP from healthy and diseased human IVDs to identify A) changes of the cellular composition in degenerated AF and NP tissue compared to non‐degenerated tissue, and B) the distinctive gene expression profiles and their key regulators during IDD.

## 2 Results

### 2.1 Disease‐Related Changes in the Cellular Landscapes of AF and NP

For this study, we obtained lumbar spines from three young healthy, and ten older donors with varying degrees of IDD (Table S1, Supporting Information). This included Thomson grade II samples (n = 3; age 21–27) which were considered “healthy”, and grades II‐III (n = 2; age 37–43), III (n = 2; age 42–63), and III‐IV (n = 6 [AF; age 59‐73]; n = 4 [NP; age 61‐68]) as “diseased” IVDs.^[^ ^20^ ^]^ We surgically separated AF and NP and following single‐cell isolation, barcoding, library preparation, and sequencing, cell clusters were analyzed for marker genes and characterized for their unique functions. The R package, Seurat, was used for Uniform Manifold Approximation and Projection (UMAP) dimensionality reduction.^[^ ^21^ ^]^

**Figure 1:**
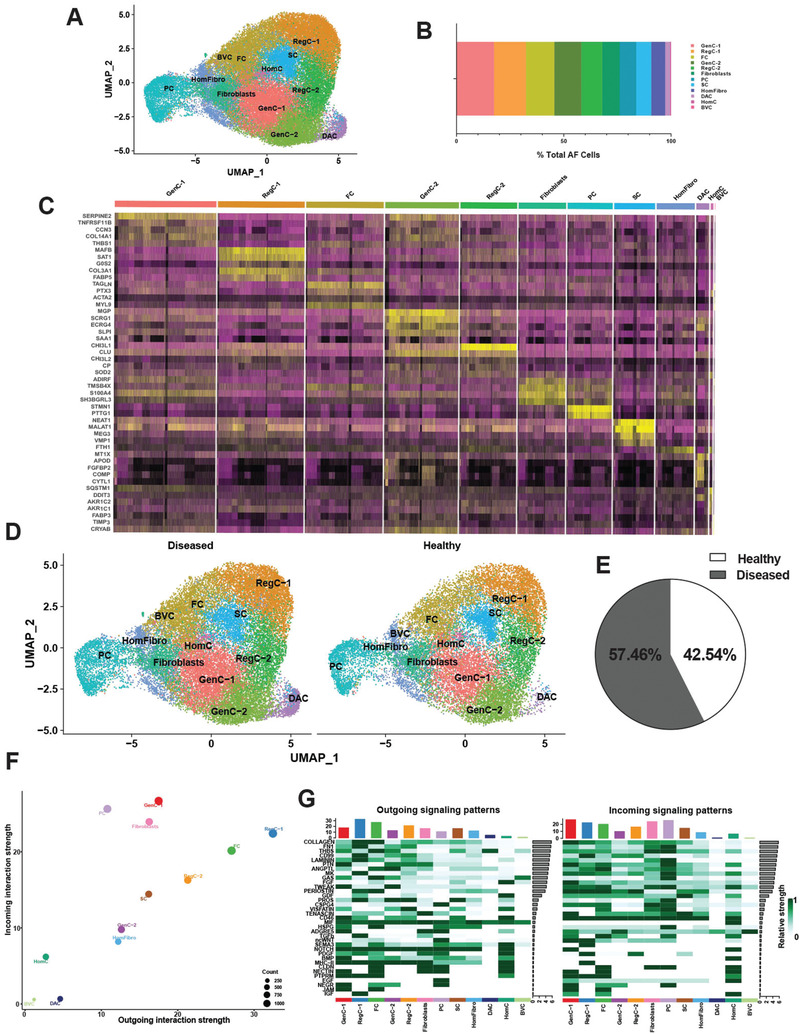
Single‐cell RNA sequencing of healthy and diseased human AF. A) Visualization of clustering by UMAP plot of healthy and diseased AF samples (n = 13) using 0.5 resolution. B) Quantification of AF cluster contribution shown as percentage of total AF cell count of the integrated data set. C) Top markers for each AF cluster visualized in a heat map. D) Visualization of clustering by split UMAP plot of healthy (n = 3) versus diseased (n = 10) AF samples. E) Percentage of cells from each condition (healthy versus diseased) are shown in a pie chart. F) CellChat analysis was performed to interrogate cell‐cell communication patterns between clusters in AF. Overall outgoing and incoming signal strength of each cluster was visualized in a scatter plot. G) Relative strength of all enriched signals across AF clusters was visualized in a heat map. For all panels: GenC (general chondrocytes), RegC (regulatory chondrocytes), FC (fibrochondrocytes), HomC (homeostatic chondrocytes), HomFibro (homeostatic fibroblasts), PC (progenitor cells), SC (stem cells), BVC (blood vessel cells) and DAC (disease‐associated chondrocytes). Healthy = grade II; Diseased = grade II‐III, grade III and grade III‐IV.

A total of 46961 scRNA‐seq profiles from AF cells were analyzed. Using 30 principal components and a resolution of 0.5 yielded 12 clusters (**Figure** **1**A). We identified multiple populations of mature chondrocytes including 2 subsets of *SOX9* ^+^ general chondrocytes (GenC‐1 and GenC‐2), 2 subsets of *CHI3L1* ^+^ *CHI3L2* ^+^ regulatory chondrocytes (RegC‐1 and RegC‐2), fibrochondrocytes (FC) and homeostatic chondrocytes (HomC). We also identified fibroblasts and homeostatic fibroblasts (HomFibro). In addition, we found immature fibroblast progenitor cells (PC) and stem cells (SC). Cells with blood vessel signature (BVC) were observed in diseased AF as was a population which we termed disease‐associated chondrocytes (DAC). GenC‐1 and RegC‐1 were the largest clusters (Figure 1B). The top markers for each of these subsets are shown in a heat map (Figure 1C). Of the 46961 AF cells, 57.46% were from diseased (26983), and 42.54% were from healthy (19978) tissues (Figure 1D,E).

CellChat interrogates cell‐cell interactions for signals sent and signals received between cells.^[^ ^22^ ^]^ RegC‐1, FC, and RegC‐2 were the strongest senders of signals, and BVC and the DAC were the weakest. GenC‐1, PC, and fibroblasts were the strongest receivers of signals, and BVC and DAC were the weakest. (Figure 1F) Thirty‐five signaling pathways were enriched across all clusters, including collagen, FN1, THBS, laminin, and tenascin (Figure 1G), which are important in ECM homeostasis.^[^ ^23^ ^]^

**Figure 2:**
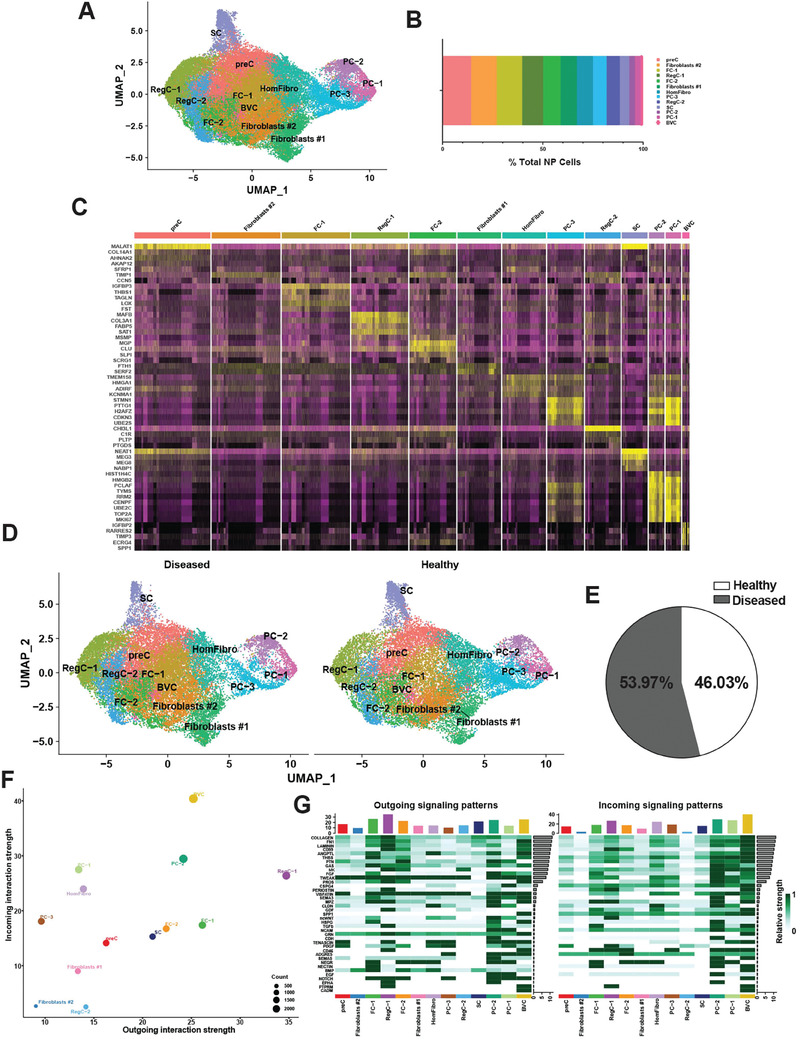
Single‐cell RNA sequencing of healthy and diseased human NP. A) Visualization of clustering by UMAP plot of healthy and diseased NP samples (n = 11) using 0.5 resolution. B) Quantification of NP cluster contribution shown as percentage of total NP cell count of the integrated data set. C) Top markers for each NP cluster visualized in a heat map. D) Visualization of clustering by split UMAP plot of healthy (n = 3) versus diseased (n = 8) NP samples. E) Percentage of cells from each condition (healthy versus diseased) are shown in a pie chart. F) CellChat analysis was performed to interrogate cell‐cell communication patterns between clusters in NP. Overall outgoing and incoming signal strength of each cluster was visualized in a scatter plot. G) Relative strength of all enriched signals across NP clusters was visualized in a heat map. For all panels: GenC (general chondrocytes), RegC (regulatory chondrocytes), FC (fibrochondrocytes), HomC (homeostatic chondrocytes), HomFibro (homeostatic fibroblasts), PC (progenitor cells), SC (stem cells) and BVC (blood vessel cells). Healthy = grade II; Diseased = grade II‐III, grade III and grade III‐IV.

In parallel to our AF studies, we also analyzed single NP cells from the same healthy (n = 3) and diseased IVDs (n = 8). A total of 45373 scRNA‐seq profiles from NP cells were analyzed. Using 30 principal components and a resolution of 0.5 yielded 13 clusters (**Figure** **2**A). We identified multiple populations of mature chondrocytes including 2 subsets of *CHI3L1* ^+^ *CHI3L2* ^+^ regulatory chondrocytes (RegC‐1 and RegC‐2) and 2 subsets of fibrochondrocytes (FC‐1 and FC‐2). In addition, we detected a population of pre‐chondrocytes (preC) that did not express stem cell markers (except for *MALAT1*), but also did not express mature chondrocyte markers. This was the largest cluster (Figure 2B). We also found 2 populations of fibroblasts as well as homeostatic fibroblasts (HomFibro). Immature fibroblast progenitor cells (PC‐1, PC‐2, and PC‐3) and stem cells (SC) were also observed. Further, the combined healthy and diseased NP cell analysis showed cells with a blood vessel signature (BVC) that was not present in healthy NP, suggesting vascular infiltration during degeneration. Unlike AF, we did not identify any uniquely disease‐associated populations in NP. The top markers for each of these subsets are shown in a heat map (Figure 2C). Of the 45373 NP cells, 53.97% were from diseased (24489), and 46.03% were from healthy (20884) tissues (Figure 2D,E).

CellChat^[^ ^22^ ^]^ analysis showed that RegC‐1, FC‐1, and FC‐2 were some of the strongest senders of signals, while BVC, all PC subsets (1, 2, and 3), and HomFibro were the strongest receivers, similar to the findings in AF. Two subsets of fibroblasts (1 and 2) and RegC‐2 were the weakest senders and receivers of signals (Figure 2F). Thirty‐nine signaling pathways were enriched across all clusters, including collagen, FN1, laminin, THBS, TGFβ, and tenascin (Figure 2G).

### 2.2 IDD‐Associated Depletion of Critical Subsets Involved in IVD Homeostasis and Self‐Renewal

**Figure 3:**
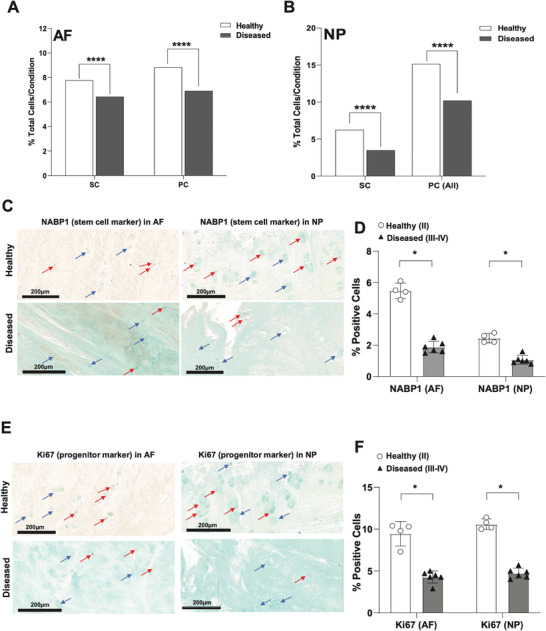
Depletion of immature subsets in diseased AF and NP. A,B) Quantification of stem cell (SC) and progenitor cell (PC) subsets in healthy versus diseased AF (A) and NP (B). Data are shown as percentage of total cells for each condition in each tissue type. ****p<0.0001 by comparison of proportions tests. Healthy = grade II; Diseased = grade II‐III, grade III and grade III‐IV. C–F) IHC for NABP1 (C,D) and MKI67 (E,F) was performed on healthy (grade II, n = 4) and diseased (grade III‐IV, n = 6) AF and NP tissues. Counterstaining was performed using methyl green to calculate percentages of positive cells verses total cell numbers. Red arrows indicate examples for positive cells and blue arrows show negative cells. Scale bars indicate 200 µm. Quantifications of cells positive for NABP1 (D) or MKI67 (F) in healthy (n = 4) versus diseased (n = 6) AF (left) and NP (right) are shown. Two‐tailed unpaired Student's *t*‐test were used to establish statistical significance. Data are expressed as means ±SD (standard deviation).*p<0.05.

We next interrogated shared changes in cellular composition in diseased IVD. In both compartments, there was a significant reduction in cells expressing markers of immature cell subsets – stem cells and fibroblast progenitors – in diseased compared to healthy IVDs (**Figure** **3**A,B). The biological processes related to stem cells in both tissue types were primarily related to RNA splicing, chromatin organization, transcription, and cell senescence (Figures S1A and S2A, Supporting Information). The biological processes related to the progenitor cells were primarily cell cycle, cell division, DNA replication, and DNA repair (Figures S1B and S2B–D, Supporting Information). These results show a loss of immature cells which are potentially required for physiological cell and tissue turnover and repair of tissue damage.

To visualize the depletion of immature cells we performed immunohistochemistry (IHC) with NABP1 as a stem cell marker (Figure 3C,D) and MKi67 (Figure 3E,F) as a progenitor marker on AF and NP tissues from healthy and diseased IVDs. By applying counterstaining with Methyl Green, the percentages of positive cells for both immature cell populations – stem and progenitor cells – were found to be significantly reduced in diseased tissue in both compartments when compared to healthy IVDs. Stem cells accounted for ≈5.4% and ≈2.6% of the total cells in healthy AF and NP, respectively (Figure 3D). Progenitor cells accounted for ≈9.8% and ≈10.8% of cells in healthy AF and NP, respectively (Figure 3F). In the diseased IVDs, the stem cell niche was reduced to ≈2.0% in AF and ≈0.8% in NP (Figure 3D), and the progenitors were reduced to ≈4.0% and ≈5.0% in AF and NP, respectively (Figure 3F).

Furthermore, in both compartments, there was a significant depletion of homeostatic fibroblasts in diseased compared to healthy (Figure S3A, Supporting Information). The biological processes of these subsets are primarily related to homeostasis mechanisms including translation and protein metabolomics, cell motility and proliferation, oxidative phosphorylation, and detoxification, and immune signaling (Figure S3B,C, Supporting Information). Depletion of these cells in both compartments indicates an impairment of cellular homeostasis during degeneration.

### 2.3 Expansion of Fibrotic Chondrocyte Subsets in Diseased NP

**Figure 4:**
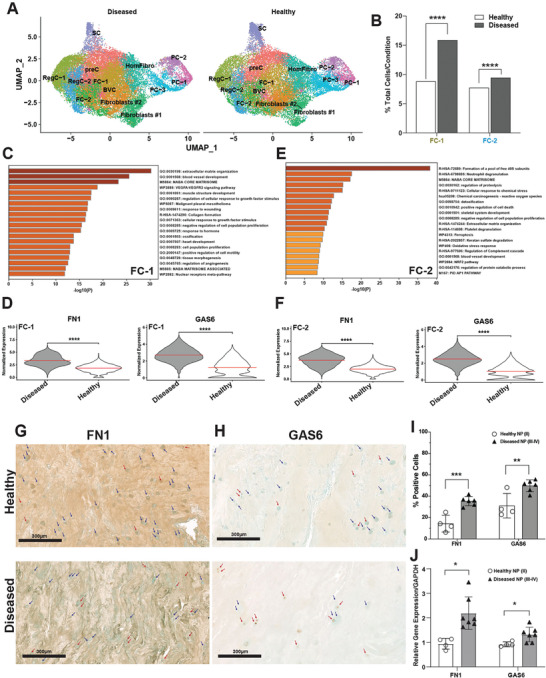
Expansion of fibrotic clusters in diseased NP. A) Visualization of clustering by split UMAP plot of healthy (n = 3) versus diseased (n = 8) NP samples. B) Quantification of FC‐1 and FC‐1 subsets in healthy versus diseased NP. Data are shown as percentage of total cells for each condition. ****p<0.0001 by comparison of proportions tests. (C,E) Metascape analysis of gene markers of FC‐1 C) and FC‐2 E). (D,F) FN1 and GAS6 are among the top DEGs in diseased NP compared to healthy in FC‐1 D) and FC‐2 F). G,H) IHC for FN1 (G) and GAS6 (H) was performed on healthy (grade II, n = 4) and diseased (grade III‐IV, n = 6) NP tissues. Counterstaining was performed using methyl green to calculate percentages of positive cells verses total cell numbers. Red arrows indicate examples for positive cells and blue arrows show negative cells. Scale bars indicate 200 µm. I) Quantifications of cells positive for FN1 and GAS6 in healthy (n = 4) versus diseased (n = 6) NP are shown. Two‐tailed unpaired Student's *t*‐test were used to establish statistical significance. Data are expressed as means ±SD (standard deviation).^**^ *p* <0.01, ^***^ *p* <0.001. J) qPCR showing FN1 and GAS6 mRNA abundance in whole healthy (grade II, n = 4) versus diseased (grade III‐IV, n = 7) NP. Data are relative to GAPDH. Two‐tailed unpaired Student's *t*‐test were used to establish statistical significance. Data are expressed as means ±SD (standard deviation). *p <0.05.

In contrast to reduction in immature cell subsets shared between the two IVD compartments, we also discovered alterations in cellular composition specific to degenerated AF and NP. In NP, both fibrochondrocyte populations (FC‐1 and FC‐2) were significantly expanded in diseased compared to healthy (**Figure** **4**A,B). The marker genes expressed in FC‐1 were primarily involved in ECM organization, blood vessel development, TGFβ signaling, wound response, and ossification (Figure 4C). Some of the top differentially expressed genes (DEGs) in FC‐1 upregulated in diseased compared to healthy were *FN1* and *GAS6* (Figure 4D). The genes involved in FC‐2 were primarily involved in immune signaling, ECM organization, oxidative stress response, and blood vessel development (Figure 4E). As in FC‐1, *FN1* and *GAS6* were among the top DEGs (Figure 4F). FN1 (fibronectin 1) has previously been reported to be associated with IVD degeneration,^[^ ^24^ ^]^ and to promote fibrosis in several other tissues.^[^ ^25^ ^]^ GAS6 also plays an important role in fibrosis^[^ ^26^ ^]^ as well as tissue remodeling and inflammation.^[^ ^27^ ^]^ We confirmed the upregulation of *FN1* and *GAS6* expression in diseased NP compared to healthy NP using IHC (Figures 4G–I) and qPCR (Figure 4J). Taken together, these data support a role of these genes in promoting fibrosis and extensive tissue remodeling in NP during disease progression (Figure S10E, Supporting Information).

### 2.4 The Fibrotic Chondrocyte Subsets are the Major Sources of Pathogenic Thbs Signaling in NP

**Figure 5:**
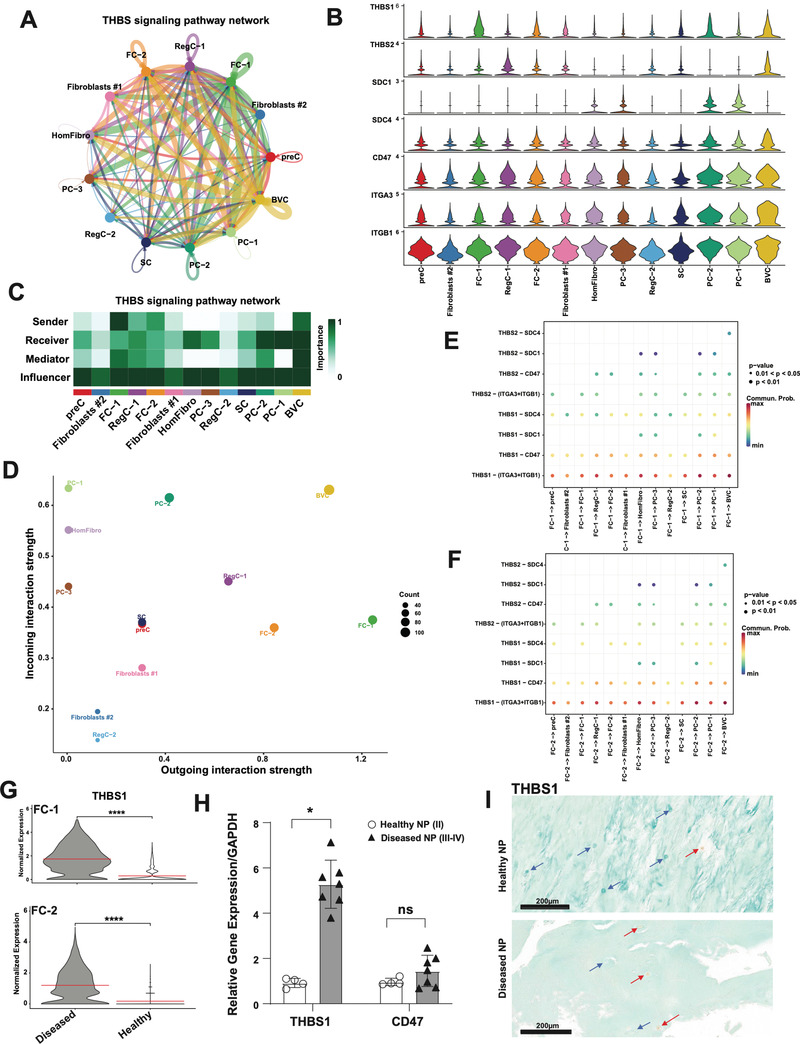
The expanded fibrotic clusters are critical regulators of THBS signaling in NP. A,C) Circle plot showing direction (A) and heat map (C) showing role importance in the four CellChat‐defined centrality measures in the THBS signaling pathway in all clusters in NP. B) Genes involved in THBS signaling network and their relative expression levels in each NP cluster. D) Overall outgoing and incoming signal strength of each cluster in the THBS signaling network visualized in a scatter plot. E,F) Significance of each ligand‐receptor (L‐R) signaling interaction comprising “THBS” signaling network in NP. FC‐1 (E) and FC‐2 (F) were set as the sources of the signal. G) THBS1 is DE in diseased NP compared to healthy in FC‐1 (top) and FC‐2 (bottom). H) qPCR showing THBS1 and CD47 mRNA abundance in healthy (grade II, n = 4) versus diseased (grade III‐IV, n = 7) NP. Data are relative to GAPDH. Two‐tailed unpaired Student's *t*‐test were used to establish statistical significance. Data are expressed as means ±SD (standard deviation). *p <0.05. I) IHC for THBS1 and staining was performed on healthy (grade II, n = 4) and diseased (grade III‐IV, n = 6) NP tissues. Counterstaining was performed using methyl green to calculate percentages of positive cells verses total cell numbers. Red arrows indicate examples for positive cells and blue arrows show negative cells. Scale bars indicate 200 µm.

To understand how the fibrochondrocyte subsets interact with the other subsets in NP, we performed CellChat^[^ ^22^ ^]^ analysis. Our original analysis (Figure 2G) suggested that the FC‐1 and FC‐2 were key senders of a variety of signals, one of which was THBS (thrombospondin) **Figure** **5**A). The gene expression network comprising the CellChat‐defined THBS signaling pathway is shown in a violin plot (Figure 5B). Across the entire signaling network, FC‐1 was the strongest sender of THBS signals, followed by BVC and FC‐2. BVC, all PC subsets, and HomFibro were all strong receivers of this signal. These data are represented in heat map and scatter plot (Figures 5C,D). Specifically, FC‐1 and FC‐2 were senders of THBS1 (Figures 5E,F). The enriched cell surface receptors of THBS1 were ITGA3, ITGB1, CD47, SDC1, and SDC4 (Figures 5E,F). *THBS1* was shown to be upregulated in diseased cells compared to healthy in both FC‐1 and FC‐2 (Figure 5G). These findings were further evaluated by performing qPCR for *THBS1* gene expression in NP tissue from healthy and diseased IVDs. The results confirmed upregulation of *THBS1* in diseased compared to healthy (Figure 5H), suggesting that this is an important pathogenic signal. *CD47* was unchanged in diseased NP compared to healthy (Figure 5H). These results were further supported by IHC analysis showing a significant increase of THBS1 positive cells in diseased NP, which had 52.1% positive cells compared to 1.7% in healthy NP (Figure 5I; Figure S4A, Supporting Information).

**Figure 6:**
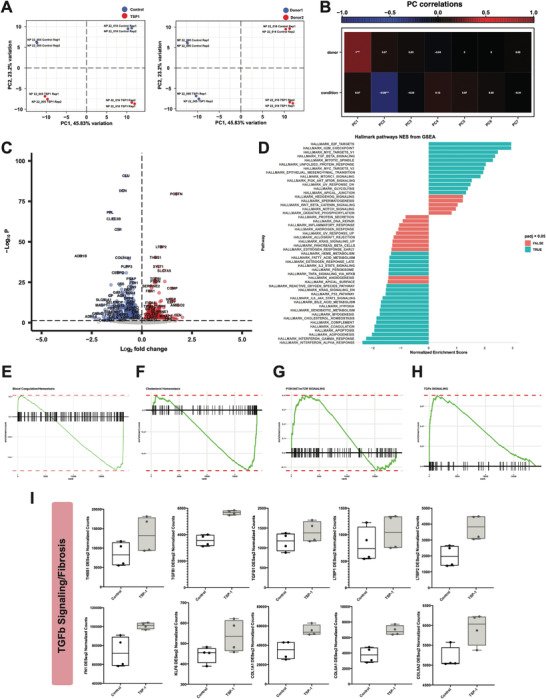
THBS is a pathogenic signal in NP. A) PCA plot showing separation by condition (left, control versus TSP‐1) and donor (right, donor 1 versus donor 2). B) Heat map showing correlations of condition and donor with each principal component. C) Volcano plot showing DEGs in control versus TSP‐1 treated NP cells. D) Hallmark gene set enrichment analysis of the DEGs. Genes were ranked by DESeq2 “stat” value (Wald statistic, log_2_FC divided by standard error). Significantly enriched pathways (padj <0.05) are indicated in blue. E–H) Enrichment plots showing significantly enriched pathways from GSEA (HALLMARK_COAGULATION; HALLMARK_CHOLESTEROL_HOMEOSTASIS; HALLMARK_PI3K_AKT_MTOR_SIGNALING; HALLMARK_TGF_BETA_SIGNALING). Enrichment plots show the gene set name (top), the running enrichment score (green curve) and the positions of the gene set hits on the rank ordered list in GSEA (black bars). I) Box plots showing DESeq2 counts for genes involved in TGFb/fibrosis signaling from control or TSP‐1 treated NP cells (n = 2 donors with n = 2 technical replicates/donors). Line at the median.

To further validate that THBS1 is a pathogenic signal in NP, we treated NP cells from 2 healthy, young donors with recombinant TSP‐1 protein and performed bulk RNA sequencing. Principal component analysis (PCA) revealed separation of samples by both condition and donor (**Figure** **6**A). Donor variation was significantly contributing to PC1, and condition was significantly contributing to PC2 (Figure 6B). Therefore, we accounted for donor variation in the DESeq2 design matrix before performing DE analysis based on condition (control versus TSP‐1 treatment, described in methods). This analysis revealed 1038 DEGs (571 downregulated genes (DRGs) and 467 upregulated genes (URGs)) (Figure 6C). Hallmark gene set enrichment analysis (GSEA) identified the MSigDB pathways enriched in the DRGs were primarily related to interferon signaling, blood coagulation, and cholesterol homeostasis (Figures 6D–F). MSigDB gene set pathways enriched in the URGs were related to cell cycle, PI3K/AKT/mTOR signaling, unfolded protein response (UPR), and TGFß signaling (Figures 6D,G,H). Some of genes upregulated involved in TGFß signaling include: *THBS1, TGFBI, TGFB1, LTBP1, and LTBP2* (Figure 6I). Several other fibrosis‐related genes upregulated by TSP‐1 treatment were: *FN1* and *KLF6* as well as fibrotic collagens including *COL1A1, COL5A1*, and *COL5A2* (Figure 6I).

### 2.5 Identification and Characterization of an Expanded Pathogenic Chondrocyte Population in Diseased AF

**Figure 7:**
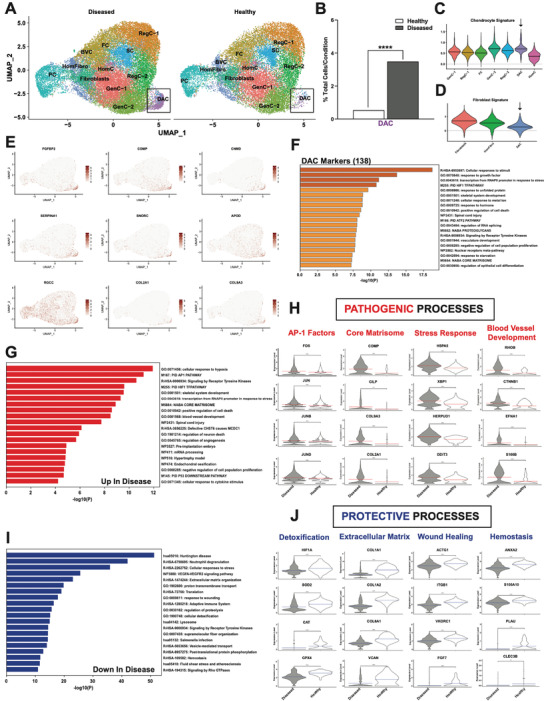
Identification of an expanded, disease‐associated chondrocyte population in AF. A) Visualization of clustering by split UMAP plot of healthy (n = 3) versus diseased (n = 10) AF samples. The disease‐associated chondrocyte (DAC) subset is shown in black boxes. B) Quantification of the DAC subset in healthy versus diseased AF. Data are shown as percentage of total cells for each condition. ****p<0.0001 by comparison of proportions tests. Healthy = grade II; Diseased = grade II‐III, grade III and grade III‐IV. (C, D) Lists of genes composing individual cluster signatures were established based on previously published literature (Table S2, Supporting Information). The Seurat command AddModuleScore was used to add a signature score to each individual cell in the integrated Seurat object based on mean expression of the genes in the signature lists. These scores were then visualized in violin plots split by cluster. Chondrocyte C) and fibroblast D) signatures are visualized for respective AF clusters. E) UMAPs showing SCT normalized counts of top markers for the DAC: *FGFBP2*, *COMP*, *CNMD*, *SERPINA1, SNORC, APOD, RGCC, COL2A1* and *COL9A3*. F) Functional enrichment analysis using Metascape of the 138 significant markers of the DAC. G,I) Metascape analyses of the genes upregulated (G) and downregulated (I) in diseased compared to healthy in the DAC. H,J) Biological processes and DEGs annotated for these processes in upregulated (H) and downregulated (J) in the DAC.

In contrast to NP where we observed significant expansion of fibrotic populations that already existed in healthy, in diseased AF we observed significant expansion of a novel disease‐associated subset (**Figure** **7**A,B). This subset had the strongest chondrocyte signature compared to other known chondrocyte populations (Figure 7C; Table S2, Supporting Information), but it did not have a fibroblast signature as seen in the other identified fibroblast populations (Figure 7D; Table S2, Supporting Information). Therefore, we termed this subset disease‐associated chondrocytes (DAC). Several of the top markers of the DAC included: *FBFBP2*, *COMP*, *CNMD*, *SERPINA1*, *SNORC*, *APOD*, *RGCC*, *COL2A1*, and *COL9A3* (Figure 7E; Figure S5, Supporting Information). Metascape^[^ ^28^ ^]^ analysis of the 138 significant gene markers of this cluster revealed enrichment of biological processes related to response to stimuli, fibrosis signaling, transcription, UPR, skeletal system development, RNA splicing, vasculature development, and ECM (Figure 7F). These processes are a mix of both immature stem cell‐like processes and mature chondrocyte‐like processes.

Although the DAC is a chondrocyte population, it was highly distinguishable from all other chondrocyte subsets (Figure S6A–F, Supporting Information). The DAC was most similar to GenC‐1 and GenC‐2, and shared *SOX9* – a master regulator of chondrocyte maturity – as a marker gene (Figure S6A,B, Supporting Information). Several key markers that differentiated the DAC from the other chondrocyte subsets included: *CYTL1, APOD, CLEC3A, CHAD, MIA, COMP, CILP, ACAN, COL2A1, COL9A2, COL9A3, TXNIP* and *FRZB*. The only exception was GenC‐2, which shared with the DAC the marker genes: *APOD, COMP, ACAN, COL9A3*, and *MIA*. Additional unique markers of GenC‐2 included: *MSMP, MGP, COL8A1, COL11A1*, and *COL14A1* (Figure S6B, Supporting Information). *CHI3L1* and *CHI3L2* were markers of both RegC‐1 and RegC‐2, but markers of RegC‐1 also included several collagens such as types I, III, V, and VI (Figure S6C,D, Supporting Information). Markers of fibrochondrocytes included types I, III, IV, V, VI, VIII, and XI collagens, in addition to several ADAM metallopeptidases with thrombospondin type 1 motif genes (*ADAMTS1*, *ADAMTS2*, and *ADAMTS5*) and matrix metallopeptidase 2 (*MMP2*) (Figure S6E, Supporting Information).

Taken together, we have identified a novel disease‐associated chondrocyte subset in AF, with highly unique gene markers and ontology programs that distinguish it from all other chondrocyte populations. Additionally, the DAC appears to maintain some stem cell ontology processes, potentially suggestive of an incomplete or abnormal differentiation or activation program.

### 2.6 Disease‐Associated Chondrocytes: Dysregulated Genes and Pathways

To investigate the genes that were altered in the DAC, we performed DE analysis. The genes upregulated in the DAC from diseased IVD compared to healthy were associated with likely pathogenic biological processes (Figure 7G,H). First, members of the AP1 transcription factor family, including FOS, JUN, JUNB, and JUND (Figure 7H) were found to be highly upregulated in the DAC of diseased tissue compared to the healthy. Upregulated FOS has been associated with the development of IDD^[^ ^29^ ^]^ and inhibition of AP1 has been shown to result in an inhibition of disc degeneration.^[^ ^30^ ^]^ Additionally, genes encoding core matrisome proteins such as *COMP*, *CILP*, *COL9A3*, and *COL2A1* were upregulated in diseased (Figure 7H). COMP has previously been shown to be a marker of cartilage breakdown, and therefore a driver of disease pathogenesis in osteoarthritis knees (reviewed in^[^ ^31^ ^]^). Further, UPR genes were upregulated in diseased including: *HSPA5*, *XBP1*, *HERPUD1*, and *DDIT3* (Figure 7H). UPR is a type of ER stress response, which when chronically active promotes proteotoxicity, inflammation, and cell death. UPR has previously been linked to IDD.^[^ ^32^ ^]^ Finally, blood vessel development has been observed in degenerated IVD (Figure S10E, Supporting Information). Several genes involved in vasculature development were upregulated in diseased compared to healthy in the DAC including *RHOB*, *CTNNB1*, *EFNA1*, and *S100B* (Figure 7H).

In contrast, the genes downregulated in the DAC from diseased IVD compared to healthy were associated with likely protective biological processes including: detoxification, ECM organization, wound healing, and hemostasis (Figures 7I,J). Detoxification genes downregulated in diseased included: *HIF1A*, *SOD2*, *CAT*, and *GPX4* (Figure 7J). Antioxidants SOD2, CAT, and GPX4 play critical roles in the removal of reactive oxygen species produced by mitochondria during oxidative phosphorylation.^[^ ^33^ ^]^ ECM organization genes were downregulated in diseased compared to healthy, including type I collagens (*COL1A1*, *COL1A2*), type VI collagens (*COL6A1*, *COL6A2*, *COL6A3*), and versican (*VCAN*) (Figure 7J). All these genes have been reported to be critical components of the IVD ECM – specifically, VCAN is one of the most abundant and critical proteoglycans in IVD – and depletion of these genes results in IDD.^[^ ^34^ ^]^ Wound healing genes such as *ACTG1*,^[^ ^35^ ^]^ *ITGB1*,^[^ ^36^ ^]^ *VKORC1*,^[^ ^37^ ^]^ and *FGF7* ^[^ ^38^ ^]^ were also significantly depleted in diseased DAC compared to healthy (Figure 7J). Finally, genes involved in hemostasis such as fibrinolytic factors *ANXA2*, *S100A10*, *PLAU*, and *CLEC3B* ^[^ ^37^, ^39^ ^]^ were significantly downregulated in diseased (Figure 7J). Repression of blood coagulation genes combined with upregulation of blood vessel development genes likely leads to the establishment of abnormal blood vessels observed in degenerated IVD^[^ ^40^ ^]^ (Figure S10E, Supporting Information).

Taken together, these data suggest that the DAC expanded in diseased AF compared to healthy is likely a pathogenic population that contributes to progression of degeneration.

### 2.7 The DAC Originates from Stem Cells and Undergoes an Incomplete or Abnormal Differentiation or Activation Program

**Figure 8:**
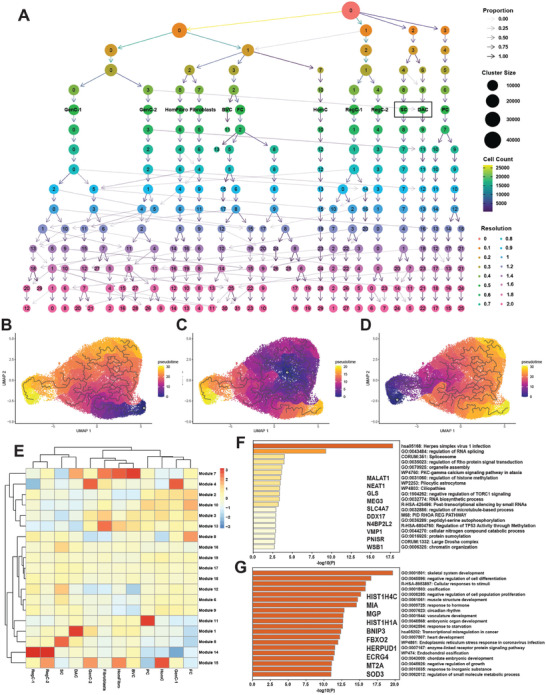
The DAC is stem cell‐derived. A) Clustree analysis showing clustering patterns from resolutions 0 through 2. Resolution 0.5 was used for all analyses. The stem cell (SC) subset and DAC are marked with black boxes. (B‐D) Monocle3 analysis revealed pseudotime trajectories with the DAC B), stem cell C) and progenitor cell D) populations set as root nodes. All AF cells were involved in reconstructing the trajectories. Root nodes are indicated by white circles. E) Heat map showing aggregated expression of modules containing genes with high variation between clusters in AF. F,G) Metascape analyses of genes in modules 5 (F) and 1 (G). Top 10 genes in each module (ranked by Moran's I) are shown.

To understand the origin of the DAC, we first performed Clustree analysis.^[^ ^41^ ^]^ Two possibilities exist: 1) the DAC originates from a pre‐existing chondrocyte population that has abnormal expression of markers, or 2) the DAC originates from stem cells and undergoes an incomplete or abnormal chondrocyte differentiation or activation program. This analysis showed a clear shared origin between the stem cell population and the DAC (**Figure** **8**A, black box). To further confirm this notion, we performed pseudotime trajectory analysis using Monocle3.^[^ ^42^ ^]^ All AF cells were involved in reconstructing trajectories. Setting the DAC as the root node of the trajectories, we observed a close transcriptomic relationship in pseudotime with the stem cell subset, but not with the progenitor cells (Figure 8B). However, setting the stem cell population as the root node, we observed a more distant relationship in pseudotime to the DAC compared to all other chondrocyte populations (Figure 8C). Additionally, setting the progenitor cells as the root node, we observed a very distant relationship with all chondrocytes and the DAC, further confirming the stem cell origin of the DAC (Figure 8D). These data confirm the stem cell origin of the DAC as suggested by Clustree analysis, while also indicating an abnormal or incomplete chondrocyte differentiation or activation program.

To explore gene expression dynamics along the trajectories, we grouped genes with high variability in expression between cell clusters into modules and visualized these modules in a heat map showing aggregated expression of each module across all cell clusters (Figure 8E). Module 5 was highly specific to the stem cell subset, but expression of this module was substantially reduced in the DAC. Metascape^[^ ^28^ ^]^ analysis of the 1038 genes in this module revealed biological processes previously characterized in stem cells including: RNA splicing, RNA biosynthesis, and chromatin organization (Figure 8F). In contrast, Module 1 was highly specific to the DAC. The 1385 genes enriched in this module were involved in more mature, chondrocytic processes including skeletal system development, ossification, and circadian rhythm (Figure 8G).

### 2.8 The DAC Retains Some Critical Regulons Specific to Stem Cells, while also having its Own Unique Regulons

**Figure 9:**
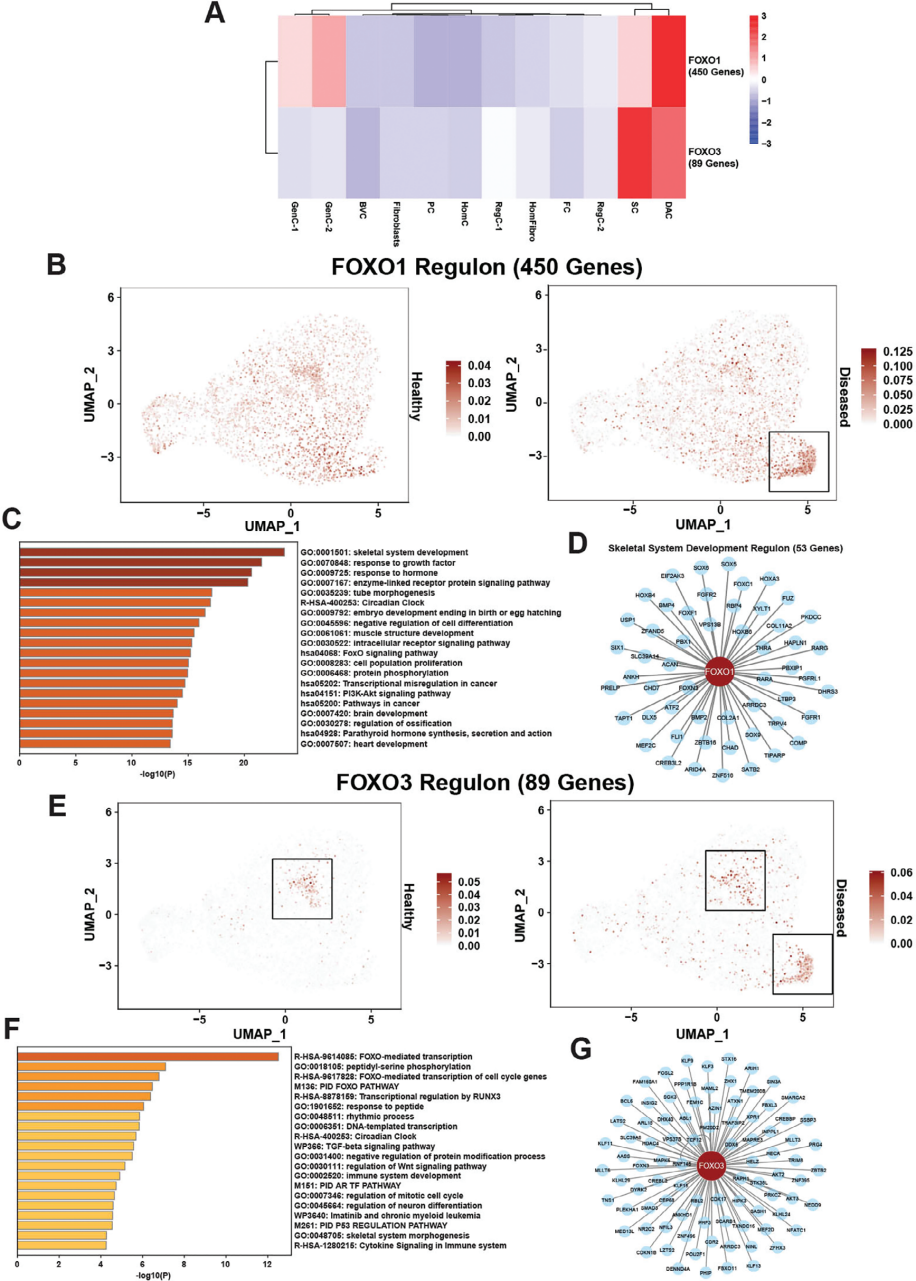
FOXO1 and FOXO3 are critical regulons in the DAC. A) pySCENIC analysis revealed FOXO1 and FOXO3 as enriched regulons in the DAC in AF. Relative regulon activity (AUC score) is shown for each regulon for each cluster in a heat map. B) FOXO1 regulon activity visualized in a split UMAP plot of healthy versus diseased AF. The DAC is shown in a black box. C) Functional enrichment analysis using Metascape of the 450 genes part of the FOXO1 regulon. D) FOXO1 skeletal system development (GO:0 001501) gene regulatory network (GRN) as visualized in a Cytoscape plot. E) FOXO3 regulon activity visualized in a split UMAP plot of healthy versus diseased AF. The stem cell subset and the DAC are shown in black boxes. F) Functional enrichment analysis using Metascape of the 89 genes part of the FOXO3 regulon. G) FOXO3 GRN as visualized in a Cytoscape plot.

To understand the gene regulatory networks (GRNs) that are dominant in the DAC compared to other AF clusters, we performed pySCENIC analysis.^[^ ^43^ ^]^ pySCENIC identifies regulons, which are composed of a regulating transcription factor (TF), and all its direct gene targets. This analysis revealed significant overlap between active regulons (AUC>1.0) in the stem cell population and the DAC (Figure S7A,B, Supporting Information). 56 regulons were unique to the DAC, one of which was FOXO1 (**Figure** **9**A). The FOXO1 regulon contained 450 genes and its DAC‐specific activity is shown in the split UMAP plot (Figure 9B). The DAC is shown in the black box. Metascape analysis^[^ ^28^ ^]^ of these genes revealed the biological processes regulated by this GRN were related to skeletal system development, fibrosis signaling, and circadian rhythm (Figure 9C). The 53 FOXO1‐regulated genes annotated with the ontology term “skeletal system development” (GO:0 001501) are shown in the Cytoscape network (Figure 9D). SOX9, a master regulator of chondrocyte differentiation, as well as its interacting partners SOX5 and SOX6 were part of this network. Additionally, critical ECM components such as COMP and COL2A1 were part of this network. Both genes were shown to be upregulated in diseased compared to healthy in the DAC (Figure 7H). Collectively, the strong representation of ECM genes which are markers of differentiation in addition to the SOX trio which regulates this process further supports the concept that this DAC is stem cell‐derived and undergoing an abnormal or incomplete differentiation or activation program.

In addition to the 56 regulons specific to the DAC, pySCENIC analysis revealed this disease‐associated population retained some critical regulons specific to stem cells (63, Figure S7A,B, Supporting Information), such as FOXO3, although at a reduced level of activity (Figure 9A). Activity of the FOXO3 regulon is shown in the split UMAP plot (Figure 9E). The FOXO3 regulon contained 89 genes, shown in the Cytoscape network (Figure 9G). The biological processes regulated by the genes in this GRN were related to transcription, cell cycle, and skeletal system morphogenesis (Figure 9F). FOXO3 was shown to play a critical role in maintenance of stem cell self‐renewal in several cell types such as muscle, blood, and neurons^[^ ^44^ ^]^; therefore, retention of some stem cell‐like regulons like FOXO3 further suggests that the DAC is stem‐cell derived and abnormally or incompletely differentiated.

### 2.9 The DAC and FC Subsets are the Major Sources of Pathogenic THBS Signaling in AF

**Figure 10:**
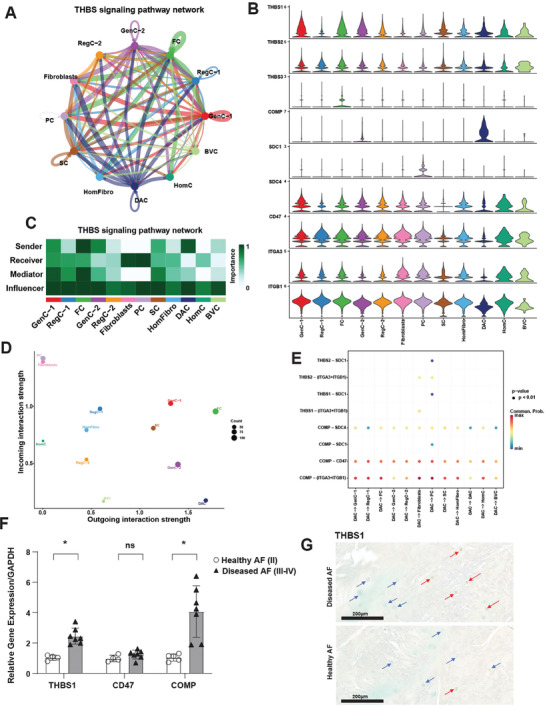
The DAC is a critical regulator of THBS signaling in AF. A,C) Circle plot showing direction (A) and heat map (C) showing role importance in the four CellChat‐defined centrality measures in the THBS signaling pathway in all clusters in AF. B) Genes involved in THBS signaling network and their relative expression levels in each AF cluster. D) Overall outgoing and incoming signal strength of each cluster in the THBS signaling network visualized in a scatter plot. E) Significance of each ligand‐receptor (L‐R) signaling interaction comprising “THBS” signaling network in AF. The DAC was set as the source of the signal. F) qPCR showing COMP, THBS1 and CD47 mRNA abundance in whole healthy (grade II, n = 4) versus diseased (grade III‐IV, n = 7) AF. Data are relative to GAPDH. Two‐tailed unpaired Student's *t*‐test were used to establish statistical significance. Data are expressed as means ±SD (standard deviation). *p <0.05. G) IHC for THBS1 and staining was performed on healthy (grade II, n = 4) and diseased (grade III‐IV, n = 6) AF tissues. Counterstaining was performed using methyl green to calculate percentages of positive cells verses total cell numbers. Red arrows indicate examples for positive cells and blue arrows show negative cells. Scale bars indicate 200 µm.

Finally, to understand how the DAC interacts with the other subsets in AF, we performed CellChat analysis.^[^ ^22^ ^]^ Our original analysis (Figure 1G) suggested that the DAC was relatively quiet – it was overall a weak sender and receiver of signals. However, the DAC was a strong sender of one key signal: THBS (**Figure** **10**A). The gene expression network comprising the CellChat‐defined THBS signaling pathway is shown in a violin plot (Figure 10B). Across the entire THBS signaling network, FC and DAC were the strongest senders of THBS signals. PC and fibroblasts were the strongest receivers of this signal. These data are represented in heat map and scatter plot (Figure 10C,D). Specifically, the DAC was a sender of both COMP and THBS1, with a greater preference for COMP‐mediated signaling (Figure 10E). The COMP and THBS1 ligands had the following receptors: ITGA3, ITGB1, CD47, SDC1 and SDC4 (Figure 10E). These data were confirmed by qPCR for *COMP* and *THBS1* showing these genes are upregulated in diseased compared to healthy AF (Figure 10F), suggesting these are important signals in pathogenesis. As in NP, *CD47* was unchanged. Interestingly, while *THBS1* is a shared pathogenic gene with NP, *COMP* is specific to AF. Furthermore, IHC confirmed a significant increase in positive cells expressing THBS1 in diseased (39.4%) versus healthy (3.3%) AF (Figure 10G; Figure S4B, Supporting Information).

To further validate that THBS1 is a pathogenic signal in AF, as before in NP, we treated AF cells from healthy, young donors with recombinant TSP‐1 protein and performed bulk RNA sequencing. As we had seen in NP, principal component analysis revealed separation of samples by both condition and donor (Figure S8A, Supporting Information). Donor variation was significantly contributing to PC1 and condition was significantly contributing to PC2 (Figure S8B, Supporting Information). Therefore, as before we accounted for donor variation in the DESeq2 design matrix before performing DE analysis based on condition. This analysis revealed 1781 DEGs (885 DRGs and 896 URGs) (Figure S8C, Supporting Information). As in NP, Hallmark GSEA identified the MSigDB pathways enriched in the DRGs were primarily related to interferon signaling, blood coagulation, and cholesterol homeostasis (Figures S8D–F, Supporting Information). MSigDB gene set pathways enriched in the URGs were related to cell cycle, PI3K/AKT/mTOR signaling, UPR, and TGFß signaling (Figure S8D,G,H, Supporting Information).

To further identify pathogenic process, pathways, and genes regulated by THBS signaling in IVD, we intersected the DEG lists from AF and NP. 411 DRGs were shared between the compartments, and 336 URGs were shared (Figure S8I, Supporting Information). Functional enrichment analysis on the 336 shared URGs using Metascape revealed enrichment of additional pathogenic processes and pathways such as nervous system development, angiogenesis, cytokine signaling, among others (Figure S8J, Supporting Information). Taken together these in vitro data suggest that thrombospondin is signaling network shared between the compartments that promotes disease progression.

## 3 Discussion

### 3.1 Major Contributions of Our Study Compared to Previous Literature

IDD is a complex and progressive process that begins early in adulthood and involves a cascade of changes on cellular, molecular, and genetic levels. scRNA‐seq allows for analysis of differential gene expression profiling of cell subpopulations within a tissue and therefore gives access to the understanding of both normal and disease‐related physiological processes that might lead to the identification of new treatment targets. Previous scRNA‐seq experiments in IDD have been performed; however, none have extensively focused on shared and compartment‐specific cellular changes in annulus fibrosus and nucleus pulposus during disease progression. Additionally, these studies have several major caveats that our studies address.

First, our study utilized larger donor numbers (n = 3 healthy and n = 10 diseased) than any previous scRNA‐seq study. Fernandes et al. (2020)^[^ ^18^ ^]^ used two healthy donors, and did not use any degenerated donors. Zhang et al. (2021)^[^ ^45^ ^]^ used one healthy and five degenerated donors. Ling et al. (2021)^[^ ^46^ ^]^ did not use any healthy donors, but used three degenerated donors. Gan et al. (2021)^[^ ^47^ ^]^ used four independent healthy donors, but analyzed five different discs in total (two discs were from the same donor). This study did not use any degenerated donors. Han et al. (2022)^[^ ^48^ ^]^ used one healthy donor and five degenerated donors (3 donors with “mild” and two donors with “severe” degeneration). Finally, Cherif et al. (2022)^[^ ^24^ ^]^ used one donor, but used 2 discs that appeared non‐degenerated and one disc that appeared degenerated from this donor.

Second, our study was the first study to use young (ages 21–27; average age: 24.3) healthy donors without a history of spine injury as a comparator to diseased samples. Only Fernandes et al. (2020)^[^ ^18^ ^]^ and Gan et al. (2021)^[^ ^47^ ^]^ utilized younger donors in their studies, but they did not compare their data to degenerated donors. Fernandes et al. (2020)^[^ ^18^ ^]^ used healthy donors of ages 24 and 35 (average age: 29.9) with no history of spinal injury or disease. In contrast, the ages of the four donors from the Gan et al. (2021)^[^ ^47^ ^]^ study were lower: 16, 31, 26, and 13 (average age: 21.5). This average age was closer to the average age of our healthy donors. However, several of the healthy donors from Gan et. al (2021)^[^ ^47^ ^]^ had a history of spinal injury or disease including: spine fracture (donor aged 16), spinal tumor (donor aged 26), and idiopathic scoliosis (donor aged 13). Outside of these two studies, the other scRNA‐seq analyses of IDD 1) did not have healthy controls, 2) used middle‐aged donors with a spinal cord injury as a healthy control, or 3) used normal‐appearing discs from the same donor that degenerated discs were obtained as healthy controls. Zhang et al. (2021)^[^ ^45^ ^]^ used 1 donor with a spinal cord injury (age 37) as a healthy control, and compared this donor to 5 donors with disc herniation (degenerated) aged 48–65. Ling et al. (2021)^[^ ^46^ ^]^ was similar to Zhang et al. (2021)^[^ ^45^ ^]^ in that 3 donors (ages 23, 32, and 44) with disc herniation were used as degenerated samples; however, this study did not use any healthy controls. Like Zhang et al. (2021),^[^ ^45^ ^]^ Han et al. (2022)^[^ ^48^ ^]^ used one healthy control with a spinal cord injury, and five donors with various degrees of degeneration. Finally, Cherif et al. (2022)^[^ ^24^ ^]^ only used one donor (age 28) in their scRNA‐seq analyses.

Third, our diseased donors spanned a wide age range and degeneration stages utilizing the Thompson grading system. We used a variety of macroscopic‐based Thompson grades^[^ ^20^ ^]^ for our diseased samples (grades II‐III, III, and III‐IV) to capture the effects of “mild” and “severe” degeneration stages, as well as to mitigate age‐related effects of the different stages of degeneration (grade II‐III average age: 40, grade III average age: ≈52; grade III‐IV average age: ≈64). Only Fernandes et al. (2020)^[^ ^18^ ^]^ and Cherif et al. (2022)^[^ ^24^ ^]^ used Thompson grading system. Fernandes^[^ ^18^ ^]^ only had healthy donors (Thompson grades I and II). Cherif^[^ ^24^ ^]^ used Thompson grades I‐II for their “non‐degenerated” discs and grade III‐V for their “degenerated” disc, but these discs originated from the same donor (age 28). All other studies^[^ ^24^, ^45^, ^46^, ^48^ ^]^ utilized the MRI‐based Pfirrmann grading system.^[^ ^49^ ^]^ Imaging‐based Pfirrmann grading system is typically less reliable than morphological‐based Thompson grading, as Pfirrmann grades tend to deviate from macroscopic assessment.^[^ ^50^ ^]^

Fourth, our single‐cell counts (NP: 45,373 cells [20,884 healthy; 24,489 diseased]; AF: 46,961 cells [19,978 healthy; 26,983 diseased]) for both tissues were higher than any other publication (excluding Gan et al. (2021)^[^ ^47^ ^]^ which had 53,165 NP cells). Fernandes et al. (2020)^[^ ^18^ ^]^ used 725 AF cells and 1,010 NP cells, for a total of 1,735 cells across two donors. Zhang et al. (2021)^[^ ^45^ ^]^ used 30,300 NP cells across 6 donors. Ling et al. (2021)^[^ ^46^ ^]^ used 36,196 NP cells across 3 donors. Gan et al. (2021)^[^ ^47^ ^]^ used 108,108 total cells from NP, AF, and cartilage endplate (CEP) tissues (NP: 53,165; AF: 27,001; CEP: 27,942) across four donors. Han et al. (2022)^[^ ^48^ ^]^ used 30,300 NP cells across six donors. Finally, Cherif et al. (2022)^[^ ^24^ ^]^ used 13,736 total cells (3,131 non‐degenerated AF; 3,092 degenerated AF; 3,867 non‐degenerated NP; 3,646 degenerated NP) from one donor.

Finally, this is the first study to separate NP and AF for scRNA‐seq analyses. The only other study that analyzed degenerated AF was Cherif et al. (2022).^[^ ^24^ ^]^ The other studies that used AF tissue were Fernandes (2020)^[^ ^18^ ^]^ and Gan (2021),^[^ ^47^ ^]^ and these studies only analyzed healthy tissue. Additionally, these studies integrated the data from all tissue types into one object. Our analyses of NP and AF tissues were performed separately – we did not integrate the datasets. All other remaining scRNA‐seq studies analyzed NP tissue only.^[^ ^45^, ^46^, ^48^ ^]^

### 3.2 Comparison of the Cellular Landscape Between the Healthy and Diseased IVD

For this scRNA‐seq study 46,961 AF cells from 13 donors and 45,373 NP cells from 11 donors, aged 21–73 years, were analyzed, and separated into healthy (Thompson Grade II) or diseased groups (Thompson Grades II‐III, III, and III‐IV) (Table S1, Supporting Information). In our donor collection we observed a clear age‐dependent correlation with IDD where the only healthy discs were found in under 30 years old donors and an increase in Thompson Grade with donor age. Age‐related changes, such as shorter lifespan of NP cells together with earlier senescence^[^ ^9^ ^]^ or increased oxidative stresses,^[^ ^51^ ^]^ and tissue damage caused by multiple stresses, like excessive manual labor, smoking, and genetic factors^[^ ^14^ ^]^ are known to promote degenerative processes in the IVD.^[^ ^9^, ^14^ ^]^ For the comparison of the healthy versus diseased cellular IVD landscape we analyzed AF and NP tissue‐specific Seurat objects, integrating cells from all Thompson grades and identified 12 and 13 clusters for AF and NP, respectively. Previous studies reported on 13 500 healthy and degenerated cells in a combined object with 14 clusters of an AF and NP mix.^[^ ^24^ ^]^ Other studies on only NP tissue compared over 30 000 cells from healthy (n = 1), mild and severely degenerated IDD patients (n = 5) and identified three major cell types: chondrocytes, endothelial cells, and macrophages with 7 subsets of chondrocytes,^[^ ^45^, ^48^ ^]^ or analyzed only NP cells from 3 IDD patients and identified six different cell types.^[^ ^46^ ^]^ The present study is thus the first to determine the single‐cell composition of separated human NP and AF in healthy IVD compared to diseased tissue, using large numbers of single‐cell profiles.

### 3.3 Disease‐Related Depletion of Critical Subsets Involved in IVD Homeostasis and Self‐Renewal

In this study we found a reduction of several subsets in AF and NP in diseased IVDs. We observed a statistically significant reduction in cell numbers of homeostatic fibroblasts which are essential for homeostasis mechanisms such as protein metabolomics, proliferation, and detoxification in diseased AF and NP when compared to healthy tissue. Further, we detected an also statistically significant reduction of cells expressing stem and fibroblast progenitor markers in diseased AF and NP. These immature cell populations are potentially required for maintaining tissue homeostasis. IHC also confirmed statistically significant depletion of immature cell expression Ki67 (PC) and NABP1 (SC) in diseased AF and NP compared to healthy. Our results are consistent with previous scRNA‐seq studies reporting a reduction of specific cell populations in degenerated IVD.^[^ ^48^ ^]^ Exhaustion of the native progenitor cells may be an important factor in IDD^[^ ^52^ ^]^ and a preclinical study suggested that stem cell therapy promotes repair of damaged IVD.^[^ ^53^ ^]^ These findings highlight the importance of the immature cell populations (both fibroblast progenitor and stem cell niches) in maintaining a healthy IVD and their potential for tissue repair. Consequently, the reduction or absence of resident stem cells and progenitors as shown in the present study is associated with tissue damage and promotes the chronic process of IDD. The present findings are based on the expression of previously defined markers of stem and progenitor cell populations. Demonstration of the stem and progenitor differentiation potential would provide a definitive characterization of these populations.

### 3.4 Thrombospondin Signaling is a Critical Pathogenic Signal in NP and Originates from Expanded Fibrochondrocyte Clusters

As part of the tissue remodeling process during IDD^[^ ^16b^ ^]^ we found, apart from the depletion of the immature cell populations in both tissue types, a significant expansion of two fibrochondrocyte subsets, FC‐1 and FC‐2. Similar results have been found in late‐stage IDD.^[^ ^46^ ^]^ A large amount of cells in diseased NP were found to be fibrochondrocyte cells (30‐40% of total NP cells) or NP cells that were mainly involved in inflammatory response.^[^ ^46^ ^]^ We also found a decrease in collagen II in both, FC‐1 and FC‐2, consistent with previous reports,^[^ ^54^ ^]^ and supporting the general notion of abnormal expansion and differentiation of chondrocyte subsets during IDD development.^[^ ^48^ ^]^ Further, our analyses demonstrate TGFβ signaling as a key element of the FC‐1 cluster. Increased TGFβ expression was observed in human IDD patients and suggested to participate in IDD,^[^ ^29^ ^]^ a notion supported in murine models of IVD aging and degeneration.^[^ ^55^ ^]^

One of our novel findings from CellChat analysis was that both fibrochondrocyte clusters were strong senders of THBS1 signals. Prior scRNA‐seq studies on degenerated IVD did not report findings of THBS1 upregulation or signaling.^[^ ^24^, ^45^, ^46^, ^47^, ^48^, ^53^ ^]^ In quantitative PCR analysis we additionally found a significant upregulation of *THBS1* gene expression in diseased NP when compared to healthy tissue, and immunohistochemistry further validated the higher abundance of THBS1 in degenerated tissue. Upregulation of *THBS1* gene expression has previously been observed in degenerated human NP cells upon treatment with a combination of CTGF and TGFβ1.^[^ ^56^ ^]^ Both, THBS1 and THBS2, are ECM proteins in the IVD and were shown to regulate expression levels of MMP2 and MMP9,^[^ ^57^ ^]^ which promote ECM degradation in IVD.^[^ ^58^ ^]^ In addition, a study of 720 women has reported that a THBS1 polymorphism is associated with osteophyte formation in lumbar spine degeneration.^[^ ^59^ ^]^ Our results also show that the level of MMP2, together with THBS1, is highly upregulated in both fibrochondrocyte clusters. Additionally, THBS1 has been shown to promote fibrosis both through TGFβ and independently of TGFβ^[^ ^60^ ^]^ and to play a major role in other fibrosis‐related diseases such as hepatitis^[^ ^61^ ^]^ and diabetes.^[^ ^62^ ^]^ These data suggest a role of THBS signaling in disease progression, and also are indicative of promotion of fibrosis and tissue remodeling, which are characteristic features of degenerated NP.

Our functional studies of TSP‐1 treated NP cells followed by bulk RNA sequencing validate the role THBS1 plays in promotion of fibrosis signaling, as well as several other pathogenic processes including PI3K/AKT/mTOR signaling, cytokine signaling, nervous system development, and angiogenesis. Interestingly, the blood vessel cell subset was one of the most important receivers of THBS signals from both FC‐1 and FC‐2 subsets – specifically through ITGA3 + ITGB1 receptors (Figure 5E,F). These data potentially indicate a role for the THBS1‐ITGA3/ITGB1 signaling interaction in promoting blood vessel infiltration into diseased NP. These data would be consistent with previous reports demonstrating ITGA3 and ITGB1 as key factors in promoting angiogenesis and vasculogenic mimicry in cancers.^[^ ^63^ ^]^

While the present results about changes in THBS1 expression in human IVD and its function in cell communication and regulating gene expression in AF and NP cells classify THBS1 as a candidate therapeutic target for IDD, additional in vivo assessment of its role in an animal model of IDD would further support this notion.

### 3.5 Emergence of a Disease‐Associated Chondrocytic Cluster in AF

We observed the presence of a disease‐associated cluster which was absent in healthy AF. This novel cluster accounted for 3.5% of cells in degenerated AF and we detected a strong chondrocytic gene expression signature as well as a mix of stem cell‐like processes and chondrocyte‐like processes, such as skeleton system development, RNA splicing, and ECM properties in this subset. The FOXO3 regulon, modulating genes involved in cell cycle and transcription, was found to be highly activated in the AF stem cell niche but less active in this subset, indicating not only a potential origin in stem cells but also a loss of stemness. FOXO3 has been shown in other cell types to play a critical role in stem cell self‐renewal; therefore, retention of this regulon in the DAC further supports that one interpretation that the DAC is stem cell‐derived. Additionally, Clustree analysis clearly confirmed a close relationship between the stem cell niche and this new cluster. Furthermore, fibroblast gene signature expression was found to be unpronounced in this DAC and pseudotime trajectory failed to show a relationship with the progenitor cell population, which argues for a new chondrocyte cluster with stem cell origin. An alternative mechanism is the abnormal activation of a chondrocyte‐like population which results in the expression of stem cell markers.

We also detected significant upregulation of disease‐associated processes, such as genes promoting blood vessel development and response to stresses while protective mechanisms like wound healing, and detoxification genes were significantly downregulated in the DAC. CellChat analysis revealed this new chondrocyte cluster, along with the fibrochondrocyte subset, to be the main sender of THBS signaling which is clearly associated with IDD.^[^ ^57^, ^59^ ^]^ As in NP, *THBS1* gene expression and protein abundance were enhanced in diseased AF when compared to healthy tissue. Further, as in NP, our functional studies of TSP‐1 treated AF cells validates the key role of thrombospondin in fibrosis signaling and other pathogenic processes. Unlike NP; however, *COMP* gene expression was also significantly enhanced in diseased AF compared to healthy. COMP and CTX‐II, a degradation product of collagen II, are known markers of IDD and serve as blood biomarkers of IDD.^[^ ^64^ ^]^ Interestingly, an upregulation of COMP was identified in Col IX‐deficient mice that developed IDD with age^[^ ^65^ ^]^ while we found next to the significant increase of COMP level also an upregulation of Col IX in the DAC in human IVD. Taken together, these data further confirm the importance of thrombospondin signaling in IDD pathogenesis, and suggest COMP as a critical player in AF specifically.

Further, we found the FOXO1 regulon to be highly active and specific in the novel chondrocytic population in diseased AF but mostly inactive in stem cells. This regulon contained the SOX chondrogenesis trio (*SOX5*, *SOX6*, and *SOX9*) as well as critical ECM components, *COL2A1* and *ACAN*. The representation of the SOX chondrogenesis trio and the ECM genes further supports the chondrogenic phenotype of the DAC. However, this FOXO1‐regulated GRN contains the pathogenic gene *COMP*, suggestive of a pathogenic role for FOXO1. Overexpression of Foxo1 was found to promote macrophage activation and furthering inflammatory response in mouse models and in vitro experiments with human NP cells showed that an overexpression of microRNA‐486‐5p inhibited FOXO1 and resulted in decreased apoptosis, less degradation of ECM, and inhibited inflammatory response.^[^ ^59^ ^]^ Taken together, our data support the idea that the DAC, like other chondrocytes, is derived from stem cells, but undergoes abnormal or incomplete differentiation leading to a retention of some stemness, but also activation of mature chondrocyte programs and a strongly pathogenic gene expression signature.

## 4 Conclusion

IDD pathophysiology and the exact mechanisms that lead to disc degeneration are yet to be discovered. In order to generate new therapeutics, the underlaying mechanisms of IDD need to be investigated and understood. The present study shows a reduction in immature cell numbers and the expansion of existing subsets in diseased NP tissue. We identified a novel disease‐specific cell cluster in diseased AF which is of stem cell origin and features activation of pathogenic processes. Additionally, we discovered the THBS signaling network as a driver of expanded fibrotic chondrocyte populations in diseased NP and in the novel DAC, supporting the importance of THBS signaling in IDD. Our data reveal new insights of both shared and tissue‐specific changes in specific cell populations in AF and NP during IDD (illustrated in Figure S9, Supporting Information). These identified mechanisms and molecules are novel and more precise targets for IDD prevention and treatment.

## 5 Experimental Section

### Tissue Donors and Sample Collection

Spine samples from postmortem donors (13 males; aged 21–73; Table S1, Supporting Information) with no recorded history of spine trauma or disc degeneration were obtained from Lifesharing under IRB approval. Lumbar spines were resected with surrounding muscles intact, wrapped in sterile gauze, and transported on ice. The specimens were washed with 70% EtOH and the discs were isolated intact from T12‐L1 (three donors), L1‐L2 (three donors), L2‐L3 (five donors), L3‐L4 (one donor) and L4‐L5 (one donor) using an Orthopedic bone saw OTS‐1 (Orthopedic Drills & Medical Devices, Covina, USA). Using a sterile scalpel, the AF and NP tissues were isolated. The inner transition zone between the NP and AF was removed to prevent mixing of the cells from the two compartments. AF and NP were separately stored on ice in sterile DPBS (Gibco #14 190 144) containing 10% calf‐serum. Under sterile conditions, the isolated NP and AF tissues were washed in 70% EtOH, weighed, and homogenized into very fine pieces for enzymatic digestion and single‐cell isolation.

### Macroscopic and Histological Assessment of IVDs

IVDs were cut to expose the center and macroscopic scoring was performed by applying the Thompson grading system.^[^ ^20^ ^]^ For the histological analysis, an approximately 1 cm slice was resected from the center of the IVD. Specimens were washed in phosphate‐buffered saline (PBS), fixed using zinc‐buffered formalin (ZFix, Anatech, Battle Creek, MI) for 1 week on a shaker, and embedded in paraffin. Four‐micrometer‐thick sections were cut and stained with Safranin O‐fast green.^[^ ^68^ ^]^ Histological scoring was performed using two systems.^[^ ^69^ ^]^

The IVD collection that was used in this study included 3 healthy donors with Thompson grade II and 2 donors with mild disc degeneration (Thompson III). Discs from 2 donors had very mild IDD but did neither fit the healthy grade II nor grade III but showed characteristics according to Thompson grading^[^ ^20^ ^]^ for both stages. Subsequently, these discs were categorized as an intermediate grade II‐III. Another 6 donors showed a more severe degeneration but did not show all grade IV features and were therefore categorized as advanced IDD grade III‐IV.

All Thompson grades and intermediate grades were additionally confirmed by evaluation of histology and the macroscopic Thompson grades and histologic scores were matched.^[^ ^69^ ^]^ Representative macroscopic and matched histologic examples of each grade are shown in Figure S10 (Supporting Information). The 3 IVDs with Thompson grade II showed in the histological assessment less than 25% of NP cells in clusters, no apoptotic cells, few micro‐fissures, no blood vessels, a clear lamellar structure of the AF, a clear NP/AF boundary, and a mostly uniform thickness of cartilaginous end plate (CEP). According to the “standardized histopathology scoring system for human intervertebral disc degeneration”^[^ ^69b^ ^]^ this was a score between 0–1. Discs that showed consolidated fibrous NP tissue, a loss of AF/NP discrimination, intensive mucinous material between AF lamellas, and early osteophyte development in the Thompson grading were considered grade III. Histology of these 2 discs revealed 25–75% of NP cells in clusters, few micro‐fissures in NP tissue, no clear discrimination NP/AF, no uniform thickness of CEP, some evidence of disrupted lamellas, and no clear boundaries between AF/CEP and therefore, were scored 1–2.^[^ ^69b^ ^]^ There were 2 discs that showed characteristics of grade II and III with small‐medium sized clusters in the NP lacunae, ≈25–50% of all NP cells in clusters, some apoptosis, some loss of Eosin staining around NP cells, micro‐fissures in lamellas, a mild loss of NP/AF discrimination, but still concentric lamellas, medium to dense cell pairs in CEP lacunae, some loss of demarcation between CEP to AF/NP, and were therefore assigned an intermediate grade II‐III with a score of 1. There were 6 discs that displayed the characteristics of ≈75% of NP cells in clusters, micro‐fissures and clefts in NP, apoptotic cells in NP, loss of Eosin staining, no clear discrimination of NP, and AF, no uniform thickness of CEP, disrupted AF lamellas, blood vessels and micro‐fissures in AF, dense pairs of clones but no cell clusters in CEP lacunae, but no evidence of apoptotic cells in CEP, cartilage erosion, were assigned a score 2,^[^ ^69b^ ^]^ and were considered as intermediate grade III‐IV in the Thompson grading system. In part due to the profound loss of cells in the NP, it was not possible to isolate RNA of sufficient quantity and quality (RIN >6) from discs with higher scores/grades, and those were excluded from this study.

### Single‐Cell Preparation and RNA‐Sequencing

About 500 mg minced NP tissue was digested in a 20 mL solution of 1.5% collagenase II (diluted with DMEM media + 50% CS) for 20 min at 37 °C on a shaker (80 rpm). After gently filtering of the solution with a 100 µm cell strainer, followed by a 40 µm cell strainer, the cell suspension was centrifuged at 1200 rpm for 5 min at RT. The pellet was resuspended in 1 mL of 1X DPBS with 10% CS and 1% Penicillin/Streptomycin/l‐Glutamine (Corning, Lot #30009066) and Antibiotic‐Antimycotic (Gibco, Life Tech, Lot #2441440), respectively and kept on ice.

About 500 mg very finely minced AF tissue was digested in 10 mL of 0.25% trypsin for 1 h. The cell suspension was then centrifuged at 1200 rpm for 5 min at RT and additionally digested by 2% type 2 collagenase for 2 h. All digestion solutions were placed on a shaker (80 rpm) at 37 °C. After the second digestion, the cell solution was very carefully filtered with a 100 µm cell strainer, followed by a 40 µm cell strainer, and centrifuged at 1200 rpm for 5 min at RT. The pellet was carefully loosened by gently tapping of the tube and slowly resuspend in 1 ml of 1X DPBS with 10% CS and 1% Penicillin/Streptomycin/l‐Glutamine (Corning, Lot #30009066) and Antibiotic‐Antimycotic (Gibco, Life Tech, Lot #2441440), respectively and kept on ice. Cell numbers and viability were assessed on a hemocytometer and a minimum of 1000 cells/µl (10^6^/mL) and a total of 200 µL with a total cell number of ≈200,000 with a viability >60% used for scRNA‐seq.

### Library Preparation and Single‐Cell RNA‐Sequencing

Single‐cell RNA‐seq was performed using the 10X Genomics Chromium technology. Briefly, cells were partitioned into droplets using the Chromium Controller with the Next GEM Single‐cell 3′ Reagent Kit v3.1 using dual indexing. Next, cells were lysed and the polyadenylated transcripts reverse transcribed to generate barcoded cDNA following the recommended 10X Genomics protocol. The quality of both the cDNA and the final libraries was assessed using the Agilent Bioanalyzer 2100 following the guidance provided by the 10X Genomics user guide. Libraries were loaded onto 100‐cycle flowcells for sequencing using the NextSeq2000 (Ilumina) and sequenced as follows: read1 = 28 cycles, i7 index = 10 cycles, i5 index = 10 cycles, read 2 = 90 cycles. 10000 cells were targeted per sample with a target of 20,000‐25,000 reads per cell.

### SCRNA‐Seq Data Pre‐Processing

Sequenced raw reads were processed using the Cell Ranger (v6.0.0, 10X Genomics) pipeline. Reads were aligned to the GRCh38 human reference genome. Filtered feature‐barcode matrices generated from Cell Ranger were exported into Seurat (v4.0.4) to generate individual Seurat objects.^[^ ^21^ ^]^ Seurat workflow was used to perform quality control of individual data sets, sample integration, dimensionality reduction, and clustering. Low‐quality cells and doublets for each object were filtered out at a 200 feature minimum count and ≥4000 feature maximum count, respectively. Maximum feature counts were determined on a donor‐to‐donor instance based on individual donor QC metrics. Cells with mitochondrial UMIs >5% were filtered out. Genes beginning with MT‐ were considered mitochondrial. Gene expression was normalized using SC transformation with expression regressed based on mitochondrial UMIs.

**Table:** 

Donor	Thompson Grade	Condition	AF Maximum nFeature_RNA	NP Maximum nFeature_RNA
SP20.002	III‐IV	Diseased	4000	NA
SP20.006	III‐IV	Diseased	6000	NA
SP21.007	II‐III	Diseased	7500	7500
SP21.011	III	Diseased	8000	9000
SP21.013	III	Diseased	6000	7500
SP21.014	II‐III	Diseased	7000	7500
SP21.015	II	Healthy	7500	6000
SP21.016	III‐IV	Diseased	9000	9000
SP21.017	III‐IV	Diseased	5000	9000
SP21.018	II	Healthy	6500	8000
SP22.001	II	Healthy	8000	7000
SP22.002	III‐IV	Diseased	8000	7000
SP22.003	III‐IV	Diseased	8000	5000

### Integration of Multiple Seurat Objects, Dimensionality Reduction and Clustering

Integration of multiple Seurat objects was achieved using the top 1000 features with the highest dispersion for each data set using the following Seurat commands: SelectIntegrationFeatures, PrepSCTIntegration, FindIntegrationAnchors, and IntegrateData. The top variable features were applied to principle component analysis (PCA). An elbow plot was used to determine the optimal number of components for dimensionality reduction. The top 30 components were used for UMAP (Uniform Manifold Approximation and Projection) dimensionality reduction. Clustering was performed with the FindClusters functions in Seurat using resolution 0.5.

### Marker Gene Analysis and Identification of Cell Types

The FindAllMarkers function in Seurat was used to identify signature genes of each cluster relative to all other clusters. The Wilcoxon test was performed on each gene, and the adjusted p‐value for statistical significance was calculated. A gene was considered a marker for a cluster if the gene was 1) positively expressed, 2) expressed in a minimum of 25% of the cells in the cluster and 3) had at least 0.25‐fold difference (log scale) between the cluster and the average of all other clusters. Functional enrichment analysis was performed on the gene markers for each cluster using Metascape.^[^ ^28^ ^]^ Cell clusters were annotated according to expression of the marker genes reported in the literature as well as the enriched biological processes identified by Metascape.

### Functional Enrichment Analysis of Cluster‐Specific Differentially Expressed Genes

Cluster‐specific DEGs between healthy and diseased cells were identified using the FindMarkers function in Seurat. “Healthy” or “Diseased” was first appended to the cluster identity for each cell in the integrated Seurat object. FindMarkers function was then used to identify DEGs between “Healthy” and “Diseased” in each cluster, with ident.1 = “Healthy_Cluster#” and ident.2 = “Diseased_Cluster#”. Genes were considered DE if the adjusted p value was <0.05. Upregulated and downregulated genes in diseased were imported separately into Metascape^[^ ^28^ ^]^ for functional enrichment analyses.

### CellChat

CellChat was used to investigate communication patterns between cell clusters.^[^ ^22^ ^]^ A standard CellChat pipeline could be found at https://github.com ^−1^ sqjin/CellChat/tree/master/tutorial. A “CellChat object” was first created from the integrated Seurat objects, which contained the cluster identity for each cell. The entirety of the human ligand‐receptor interaction list curated in the CellChat database (CellChatDB) was used for cell‐cell communication analysis. CellChatDB contains 1939 validated interactions, 61.8% were paracrine/autocrine signaling (“Secreted Signaling”), 21.7% were extracellular matrix (ECM)‐receptor interactions, and 16.5% were “cell‐cell contact” interactions. the overexpressed ligands and receptors in each cell cluster were next identified, followed by the determination of the overexpressed ligand‐receptor interactions. Cell–cell communication probabilities for each ligand‐receptor interaction were inferred using permutation tests. The inferred communication probability values were then visualized in circle and scatter plots. Importance of each cell cluster in the signaling pathway of interest was also computed, and represented in heat maps in four CellChat‐defined centrality measurements (sender, receiver, mediator, and influencer). Cluster‐specific expression of all genes comprising the signaling pathway of interest was shown in violin plots. The most significant ligand‐receptor pairings for each signaling pathway of interest were also visualized in a bubble plot. These follow‐up analyses on the “THBS” pathway were promoted.

### Monocle3

To determine differentiation trajectories governing cellular hierarchy in AF and NP cells, Monocle3 analyses were performed.^[^ ^42^ ^]^ All AF and NP cells were involved in reconstructing the trajectories using Monocle3. Root nodes were set in the stem cell, progenitor cell, and DAC clusters, and pseudotime trajectories were calculated from each root node. To explore gene expression dynamics along the trajectories, genes with high variability between clusters were grouped into modules. These modules were visualized in a heat map showing aggregated expression of each module across all cell clusters in both tissue types. Functional enrichment analysis of the module genes was performed using Metascape.^[^ ^28^ ^]^

### pySCENIC

Python implementation of Single‐Cell reEgulatory Network Inference and Clustering (pySCENIC) was used to identify gene regulatory networks (GRNs) that govern cell types in AF and NP tissues.^[^ ^43^ ^]^ A soft filtering of genes was first applied to remove background noise. Genes were retained if they were expressed at a log_2_FC of 3 in at least 1% of the cells. In AF, 9773 genes were retained and in NP 9613 genes were retained for downstream analyses. The filtered expression matrices were then exported for Python‐based analyses using the command exportsForArboreto as part of the SCENIC R package. GRN analysis was first performed on these expression matrices using GRNBoost2, which identified transcription factors (TF) and target genes with significant co‐expression patterns. The motif databases (hg19‐500bp‐upstream and hg19‐tss‐centered‐10 kb) were used for RcisTarget analysis, which created modules in which the binding motif of the regulating TF was significantly enriched across the target genes. These resulting “regulons” consisted of a regulating TF and all its direct gene targets. Finally, AUCell was used to calculate regulon activity scores in each cell, and these scores were integrated into the metadata of the Seurat object. Cluster‐specific regulon scores were then visualized in heat maps or UMAPs. The transcriptional network of a TF and predicted target genes were visualized in Cytoscape (v3.8.2). Functional enrichment analysis of the regulon genes was performed with Metascape.^[^ ^28^ ^]^

### RNA Isolation from NP and AF Tissues and Gene Expression Analysis

Total RNA from NP and AF from lumbar discs were isolated separately. RNA from AF tissue was collected by homogenizing 100 mg tissue in 1 ml TRIzol reagent (Invitrogen, Carlsbad, CA, USA) using the Precellys 24 tissue homogenizer (Bertin Instruments, Montigny‐le‐Bretonneux, France), followed purification with two chloroform extractions. The clear phase was recovered, and RNA was isolated using the RNeasy Plus Universal Kit (Qiagen, Hilden, Germany). Total RNA from NP tissue was isolated by grounding 150 mg frozen tissue into a fine powder using a mortar and pestle and RNA was isolated using RNeasy Maxi Kit (Qiagen, Hilden, Germany).

Gene expression was measured by real‐time PCR using predesigned TaqMan gene expression assays (ThermoFisher Scientific Inc., USA) for human FN1, GAS6, THBS1, CD47, and COMP. GAPDH was used as reference gene.

### Thrombospondin (TSP‐1) Recombinant Protein Treatment

Primary human NP and AF cells were plated in duplicate in a 24‐well plate at a density of 50000 cells/well. These cells were incubated overnight and then stimulated with 100 ng of recombinant human TSP‐1 (R&D Systems, Catalog#: 3074‐TH). 24 h post‐stimulation, RNA was extracted using standard TRIzol methods.

### Bulk RNA sequencing

Total RNA was isolated and RNA quality was confirmed using NanoDrop measurements of OD 260/280 and 260/230. JC (JumpCode) libraries were generated for all replicas and the libraries sequenced on Illumina NextSeq 2000 instrument (Illumina, San Diego, CA) at an estimated 5 million reads per sample. Quality control of the fastq files was performed using FastQC (v0.12.1). Illumina 3′ small adapter sequences were trimmed from the fastq files using TrimGalore! (v0.6.10). Trimmed reads were aligned to the human genome (hg38, fasta version 110) using hisat2 (v2.2.1). Genomic features were assigned to each sample using the featureCounts function of the R Subread package (v2.14.2). Differential expression was determined using DESeq2 (v1.40.2). Donor‐to‐donor variation was accounted for in the design matrix of DESeq2. The resulting p values were adjusted using the Benjamini‐Hochberg's approach for controlling the false discovery rates (FDR). Genes with an FDR <0.05 were considered statistically significant.

### Immunohistochemistry (IHC)

IHC was performed on L1/L2 and L4/L5 from human IVD samples as described before.^[^ ^68^ ^]^ In short, 4‐mm‐thick sections were deparaffinized, rehydrated, and incubated in a water bath (for anti‐MKI67 and anti‐NABP1 treatment) for 60 min at 37 °C. The sections were blocked with 2.5% horse serum for 1 h at room temperature, followed by an overnight incubation with primary antibodies against MKI67 (1:500, Abcam, Cambridge, MA, Abcam ab15580), NABP1 (1:500, OriGene Technologies, Inc., Rockville, MD, TA811088S), CENPF (1:200, GeneTex, GTX70137), COMP (1:50, AbCam, Cambridge, MA, Abcam ab 11 056) and THSB1 (1:25, Life Technologies, NY, USA, MA513398) at 4 °C. After washing, sections were incubated with ImmPRESS DAB Reagent (Vector Laboratories, Burlingame, CA) for 30 min at room temperature followed by a 5 min incubation with DAB Peroxidase (HRP) Substrate Kit (Vector Laboratories). Counterstain was done with methyl green. Positive cells were quantified in three fields of 1mm^2^ in NP and AF. Data were expressed as the percentage of positive cells relative to the total number of cells. Cells that could not be accurately scored were not counted and were not considered in the total number of cells.

### Statistical Analysis

The processing details of the scRNA‐seq datasets were described above in the sub‐sections “scRNA‐seq data pre‐processing” and “Integration of multiple Seurat objects, dimensionality reduction, and clustering”. For AF scRNA‐seq studies, n = 3 healthy and n = 10 diseased samples were used (Table S1, Supporting Information). For NP scRNA‐seq studies, n = 3 healthy and n = 8 diseased samples were used (Table S1, Supporting Information). GraphPad Prism version 9.4.0 (GraphPad Software, San Diego, CA) was used to analyze the qPCR and quantitative IHC results by applying two‐tailed unpaired Student's *t*‐test to establish statistical significance. All quantitative data were expressed as means ±SD (standard deviation). The n values described in the legends represent biologically independent samples. For qPCR data, n = 4 healthy and n = 7 diseased samples (AF or NP) were used. For IHC data, n = 4 healthy and n = 6 diseased samples (AF or NP) were used. For macroscopic images and IHC micrographs, representative images in each group were displayed. For all statistical analyses, p‐values below 0.05 were considered significant.

## Conflict of Interest

The authors declare no conflict of interest.

## Author Contributions

H.S. and J.M. contributed equally to this work. M.K.L. designed the study. J.M. and K.M. processed tissues. J.M. isolated single‐cells. H.S., P.N. and J.M. analyzed the data. J.M., M.O., O.A‐G., S.R.H., and T.S.M. provided expertise and/or materials and methodology. K.M. and M.O. performed IHC. M.K.L. supervised the project. H.S. J.M., and M.K.L. drafted the paper, which was approved by all co‐authors.

## Supporting information

Supporting Information

## Data Availability

All data relevant to the study are included in the article or uploaded as online supplemental information. The GEO accession numbers for all the datasets utilized in the present study are GSE229711 (healthy scRNA‐seq) and GSE230808 (diseased scRNA‐seq). These datasets are connected under the super‐series GSE230809.
