# Supplementary material for: Shared and Compartment‐Specific Processes in Nucleus Pulposus and Annulus Fibrosus During Intervertebral Disc Degeneration
Source: Adv Sci (Weinh). 2024 Feb 25;11(17):2309032. doi: 10.1002/advs.202309032 (PMC11077672; doi:10.1002/advs.202309032)
Supplement: Supplementary file 1 — Supporting Information [file ADVS-11-2309032-s001.pdf]

ADVANCED SCIENCE
Open Access

Supporting Information

for *Adv. Sci.*, DOI 10.1002/advs.202309032

Shared and Compartment-Specific Processes in Nucleus Pulposus and Annulus Fibrosus During Intervertebral Disc Degeneration

*Hannah Swahn, Jasmin Mertens, Merissa Olmer, Kevin Myers, Tony S. Mondala, Padmaja Natarajan, Steven R. Head, Oscar Alvarez-Garcia and Martin K. Lotz**

SUPPLEMENTARY FIGURES

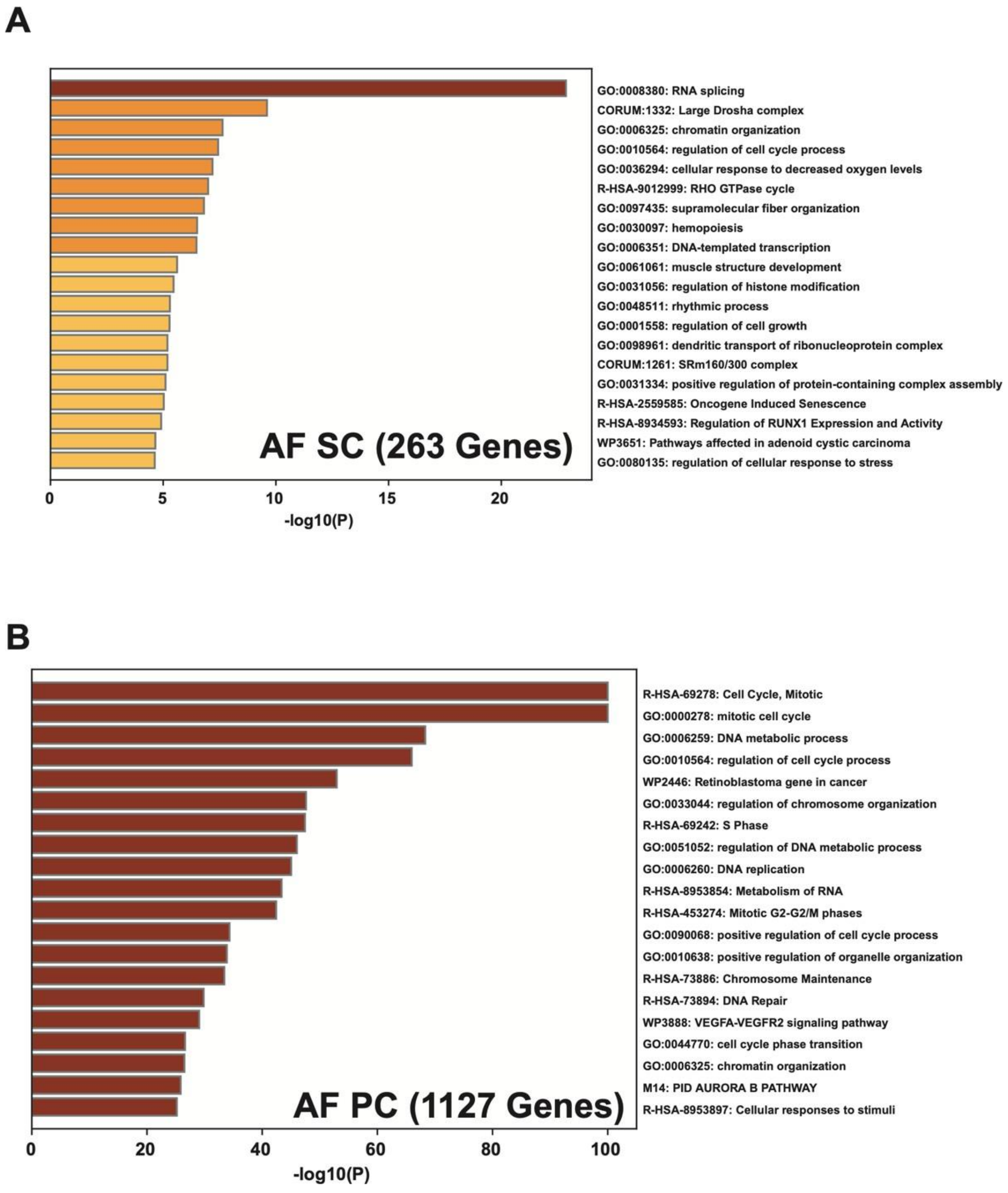

Figure S1. Functional enrichment analyses of immature populations in annulus **fibrosus (AF).** Functional enrichment analyses using Metascape of stem cells (SC) **(A)** and progenitor cells (PC) **(B)** in AF.

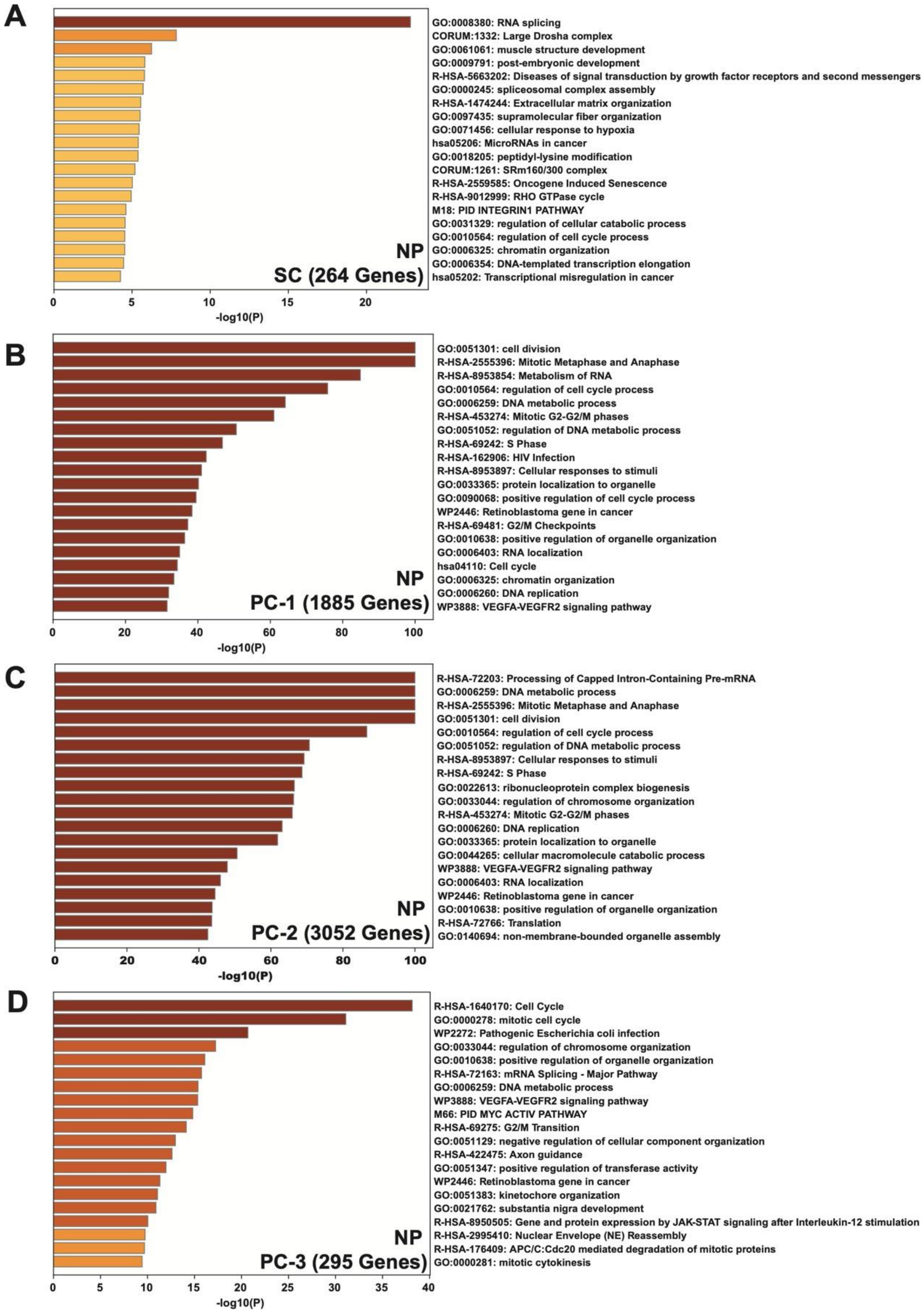

Figure S2. Functional enrichment analyses of immature populations in nucleus **pulposus (NP).** Functional enrichment analyses using Metascape of stem cells (SC) **(A)** and progenitor cells (PC) **(B-D)** in NP.

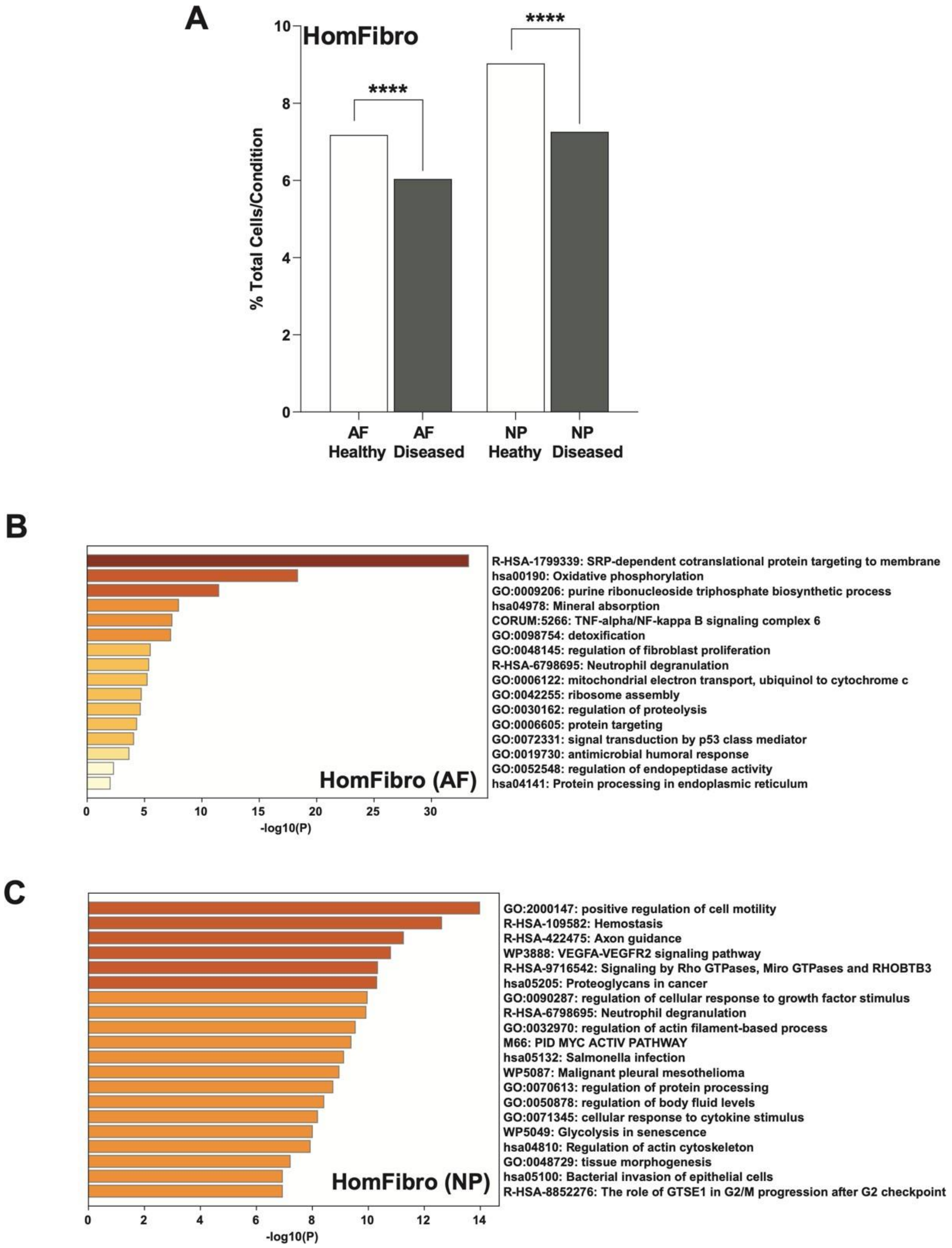

Figure S3. Depletion of the homeostatic subset in diseased AF and NP. (A) Quantification of homeostatic fibroblasts (HomFibro) in healthy vs diseased AF (left) and NP (right). Data are shown as percentage of total cells for each condition in each tissue type. ****$p<0.0001$ by comparison of proportions tests. Healthy = grade II; Diseased = grade II-III, grade III and grade III-IV. **(B, C)** Functional enrichment analyses using Metascape of gene markers of HomFibro in AF **(B)** and NP **(C).**

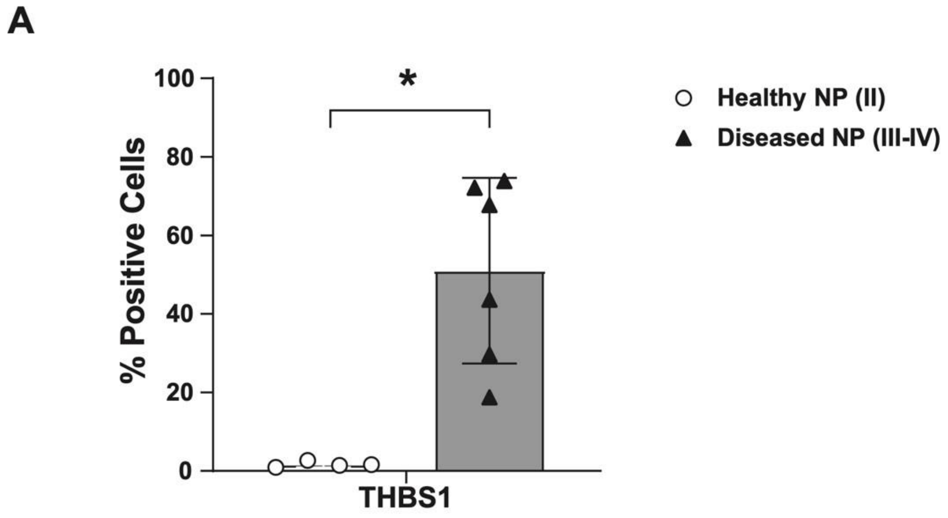

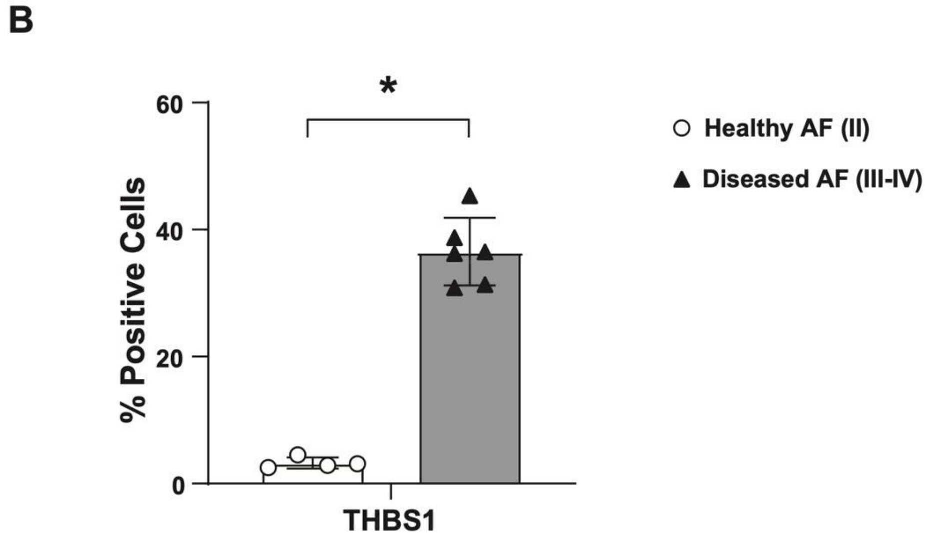

Figure S4. Immunohistochemistry of THBS1 expression in NP and AF. (A, B). Quantification of THBS1 in healthy vs diseased NP **(A)** and AF **(B)**. Data are shown as percentage of positive cells for each condition in each tissue type. ****$p<0.0001$ by comparison of proportions tests. Healthy = grade II (n=4); Diseased = grade II-III, grade III and grade III-IV (n=6).

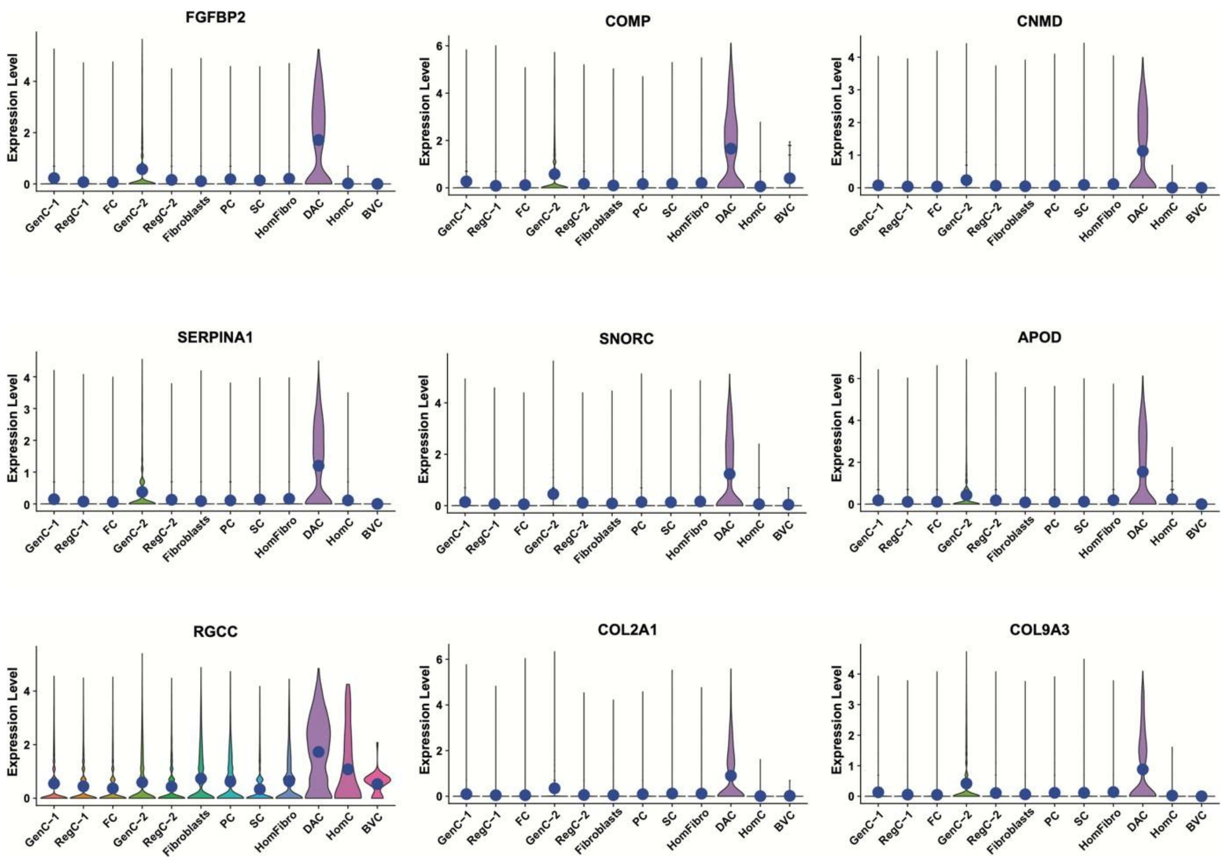

Figure S 5. Top marker genes in the disease-associated chondrocyte (DAC) subset in AF.

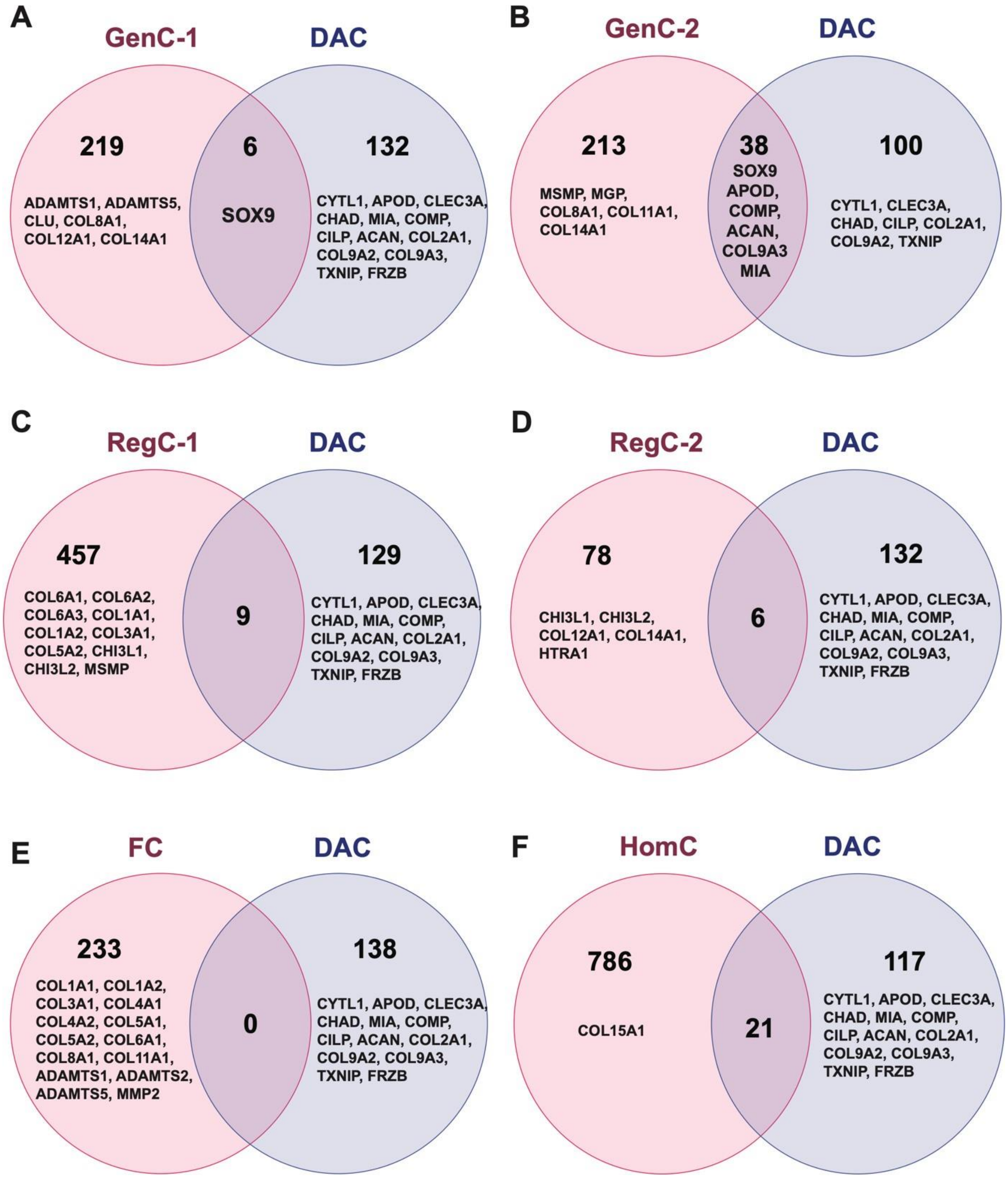

Figure S 6. Chondrocyte markers distinguishing the DAC from all other chondrocyte populations in AF. (A-F) Intersection of markers in the DAC with GenC-1 **(A)**, GenC-2 **(B)**, RegC-1 **(C)**, RegC-2 **(D)**, FC **(E)** and HomC **(F)**. Chondrocyte markers distinguishing the populations are indicated.

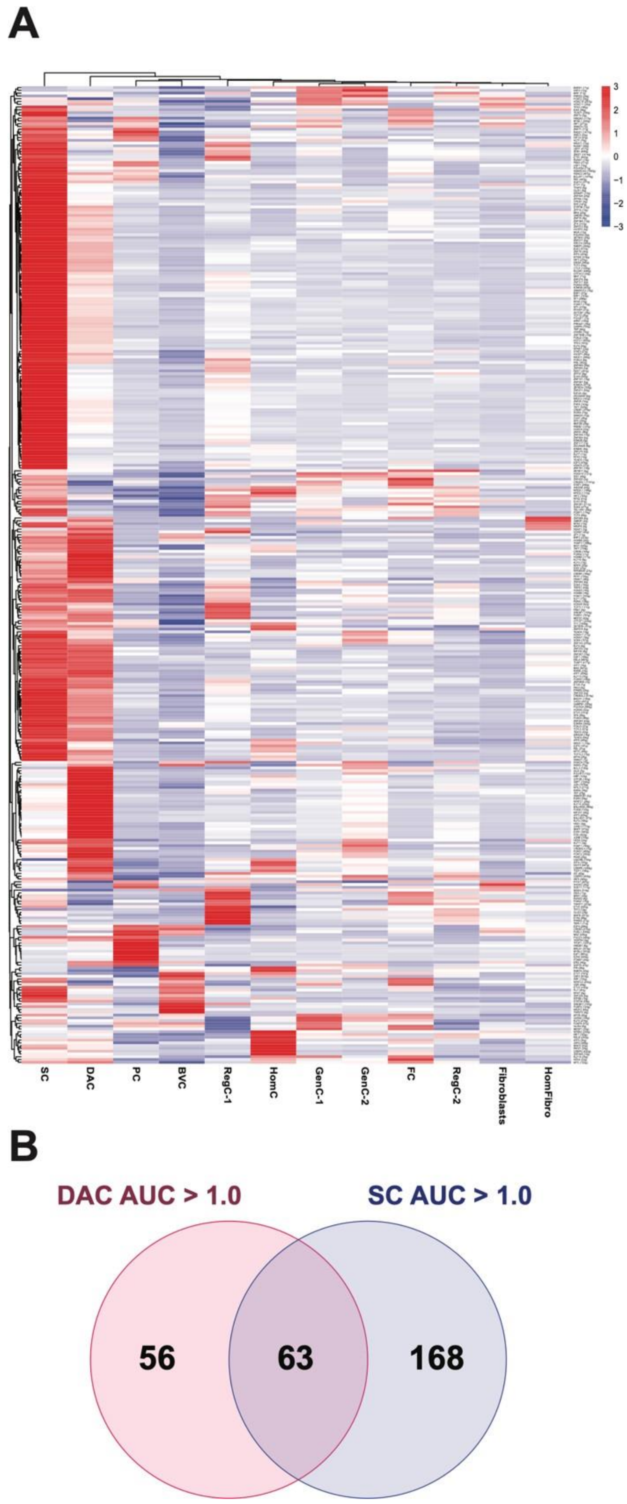

Figure S7. The DAC maintains some stem cell-specific regulons, while also gaining activity of new regulons. (A) pySCENIC analysis identifies regulons enriched across all clusters in AF. Regulon activities are visualized in a heat map. **(B)** Intersection of the active regulons (AUC > 1.0) in the DAC with the stem cell (SC) subset.

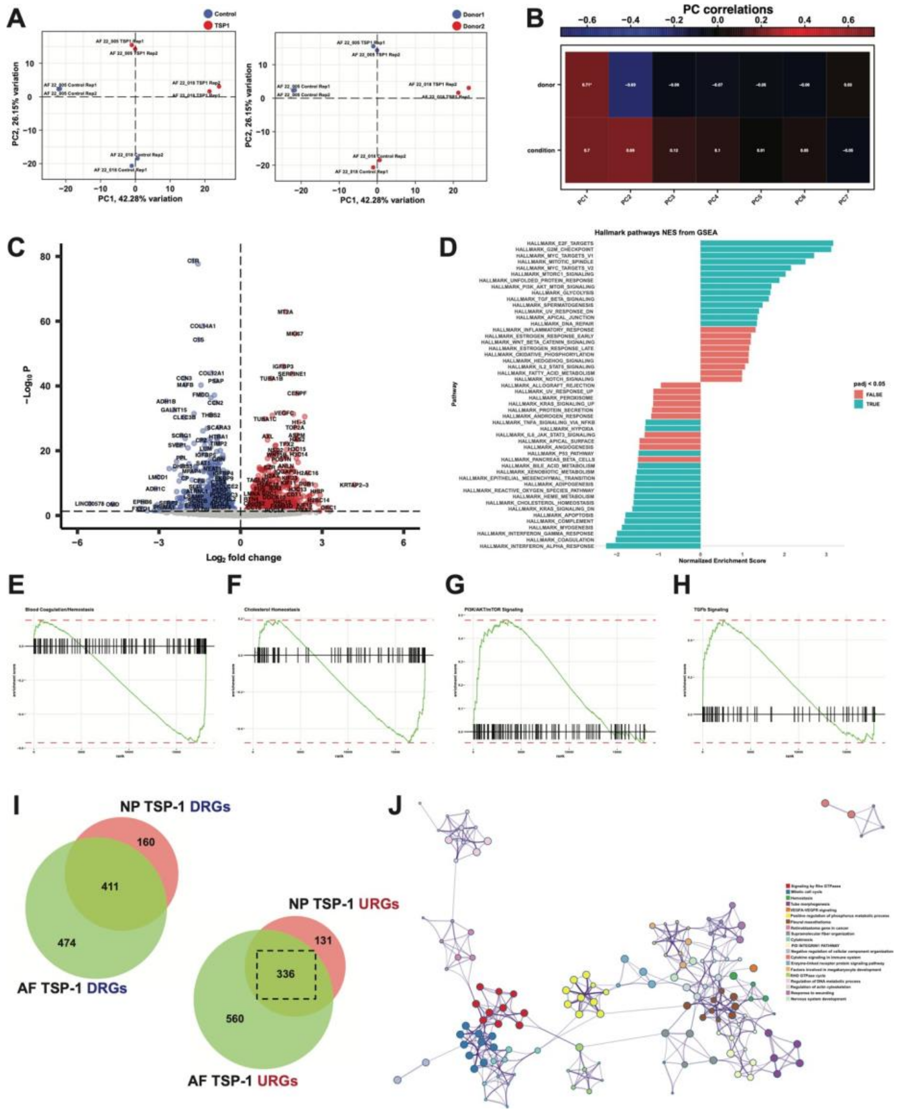

Figure S8. THBS is a pathogenic signal in AF. (A) PCA plot showing separation by condition (left, control vs TSP-1) and donor (right, donor 1 vs donor 2). **(B)** Heat map showing Pearson correlations of condition and donor with each principal component. **(C)** Volcano plot showing DEGs in control vs TSP-1 treated AF cells. **(D)** Hallmark gene set enrichment (GSEA) analysis of the DEGs. Genes were ranked by DESeq2 'stat' value (Wald statistic, log2FC divided by standard error). Significantly enriched pathways (padj <0.05) are indicated in blue. **(E-H)** Enrichment plots showing significantly enriched pathways from GSEA (HALLMARK_COAGULATION; HALLMARK_CHOLESTEROL_HOMEOSTASIS; HALLMARK_PI3K_AKT_MTOR_SIGNALING; HALLMARK_TGF_BETA_SIGNALING). Enrichment plots show the gene set name (top), the running enrichment score (green curve) and the positions of the gene set hits on the rank ordered list in GSEA (black bars). **(I)** Venn diagrams showing intersection of upregulated (URGs) and downregulated (DRGs) genes in TSP-1 treated AF and NP cells. Venn diagrams were generated using BioVenn.com. **(J)** Metascape clustering analysis of the top 20 significantly enriched biological pathways and processes regulated by the 336 URGs shared between TSP-1 treated AF and NP cells.

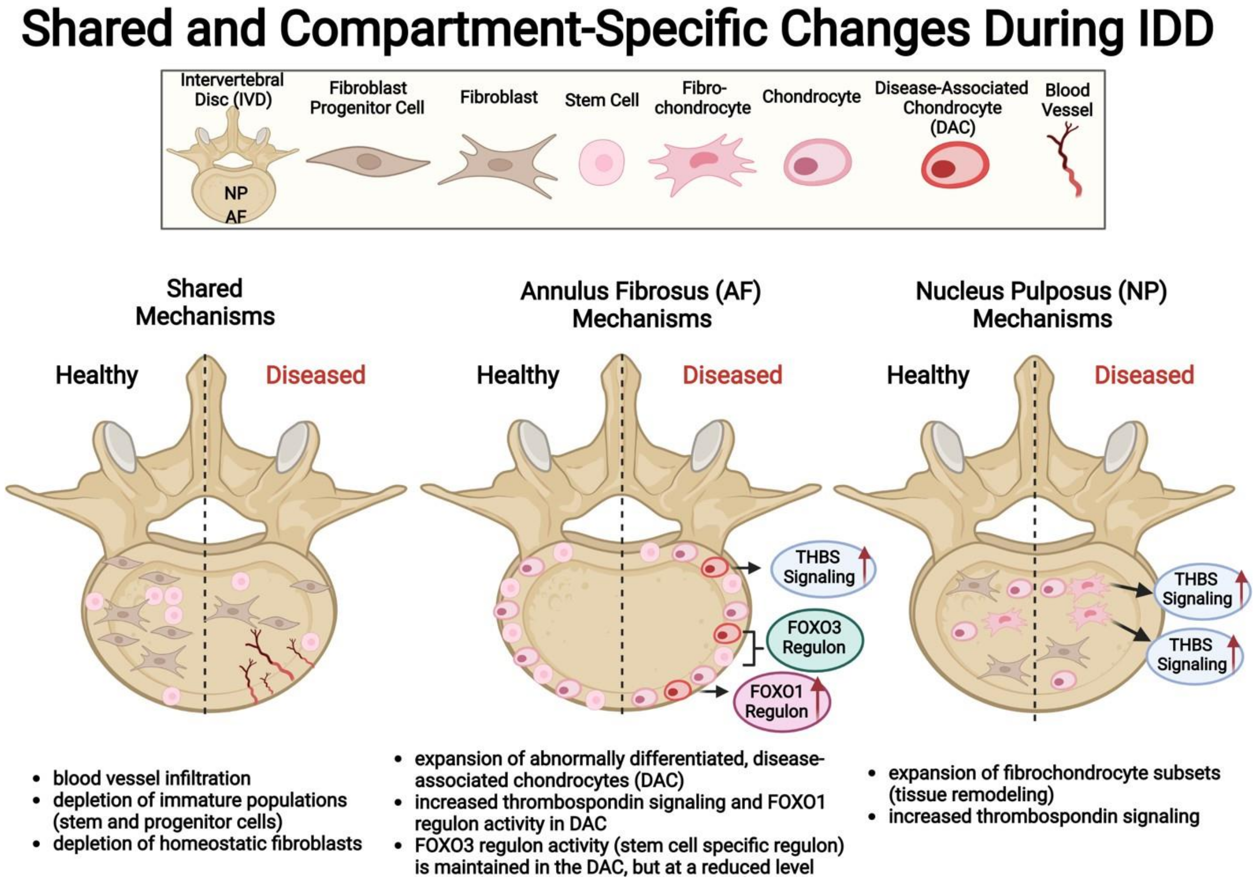

Figure S9. Schematic summary of shared and compartment specific changes during IDD. Created with BioRender.com.

A

	SP21.004	SP21.015	SP21.011	SP21.017
Thompson Grade	**II**<br>-white fibrous NP tissue<br>-clear boundary AF/NP<br>-mucinous material between lamellas	**II-III**<br>-white fibrous NP tissue<br>-not so clear boundary AF/NP<br>-mucinous material between lamellas	**III**<br>-consolidated fibrous NP tissue<br>-loss of AF/NP discrimination<br>-intensive mucinous material between lamellas<br>-early osteophyte development	**III-IV**<br>-clefts in fibrous NP tissue<br>-loss of AF/NP discrimination<br>-disruptions/loss in AF structure<br>-early osteophyte development
Rutges Score	**1-2**<br>-endplate homogeneous structure/clear regular thickness<br>-half ring AF structure<br>-clear boundary between AF/NP<br>-mixed NP cellularity with very few cell clusters<br>-largely organized NP structure<br>-red NP staining<br>-mixed red/blue AF staining	**3-4**<br>-endplate irregular thickness<br>-half ring AF structure<br>-clear boundary between AF/NP-mixed NP cellularity with clear cell clusters<br>-partly disorganized NP structure<br>-red NP staining<br>-mixed red/blue AF staining	**5-6**<br>-endplate irregular<br>-microfractures<br>-no clear half ring AF structure<br>-loss of boundary between AF/NP<br>-mixed NP cellularity with many cell clusters<br>-largely organized NP structure<br>-red NP staining<br>-mixed red/blue AF staining	**7**<br>-endplate irregular<br>-microfractures<br>-disruptions in AF structure<br>-loss of boundary between AF/NP<br>-mixed NP cellularity with many cell clusters<br>-disorganized NP structure<br>-mixed red/blue NP staining<br>-mixed red/blue AF staining
ORS Score	**0-1**<br>-single cells in lacunae<br>-no apoptotic cells<br>-<25% cells in clusters<br>-maybe few micro fissures in NP<br>-few micro fissures<br>-no blood vessels<br>-clear lamellas<br>-clear boundary NP/AF<br>-(mostly) uniform thickness of CEP	**1-2**<br>-small to medium clusters in the lacunae<br>-about 25-50% clusters<br>-some apoptosis<br>-some eosin staining loss around NP cells<br>-micro fissures in lamellas<br>-started loss of NP/AF boarder still concentric lamellas<br>-mixed cell morphology<br>-medium to dense pairs in CEP lacunae<br>-some loss of demarcation between CEP to AF/NP<br>-no uniform CEP anymore	**1-2**<br>-25-75% cells in clusters<br>-maybe few micro fissures in NP<br>-no clear discrimination NP/AF<br>-no uniform thickness of CEP<br>-some evidence of disrupted lamellas<br>-no clear boundaries AF/CEP	**2**<br>-<75% cells in clusters<br>-micro fissures and clefts in NP<br>-apoptotic cells in NP<br>-loss of eosin staining<br>-no clear discrimination NP/AF<br>-no uniform thickness of CEP<br>-disrupted lamellas<br>-blood vessels and micro fissures in AF<br>-dense pair of clones but no cell clusters in CEP lacunae<br>-no evidence of apoptotic cells in CEP<br>-cartilage erosion

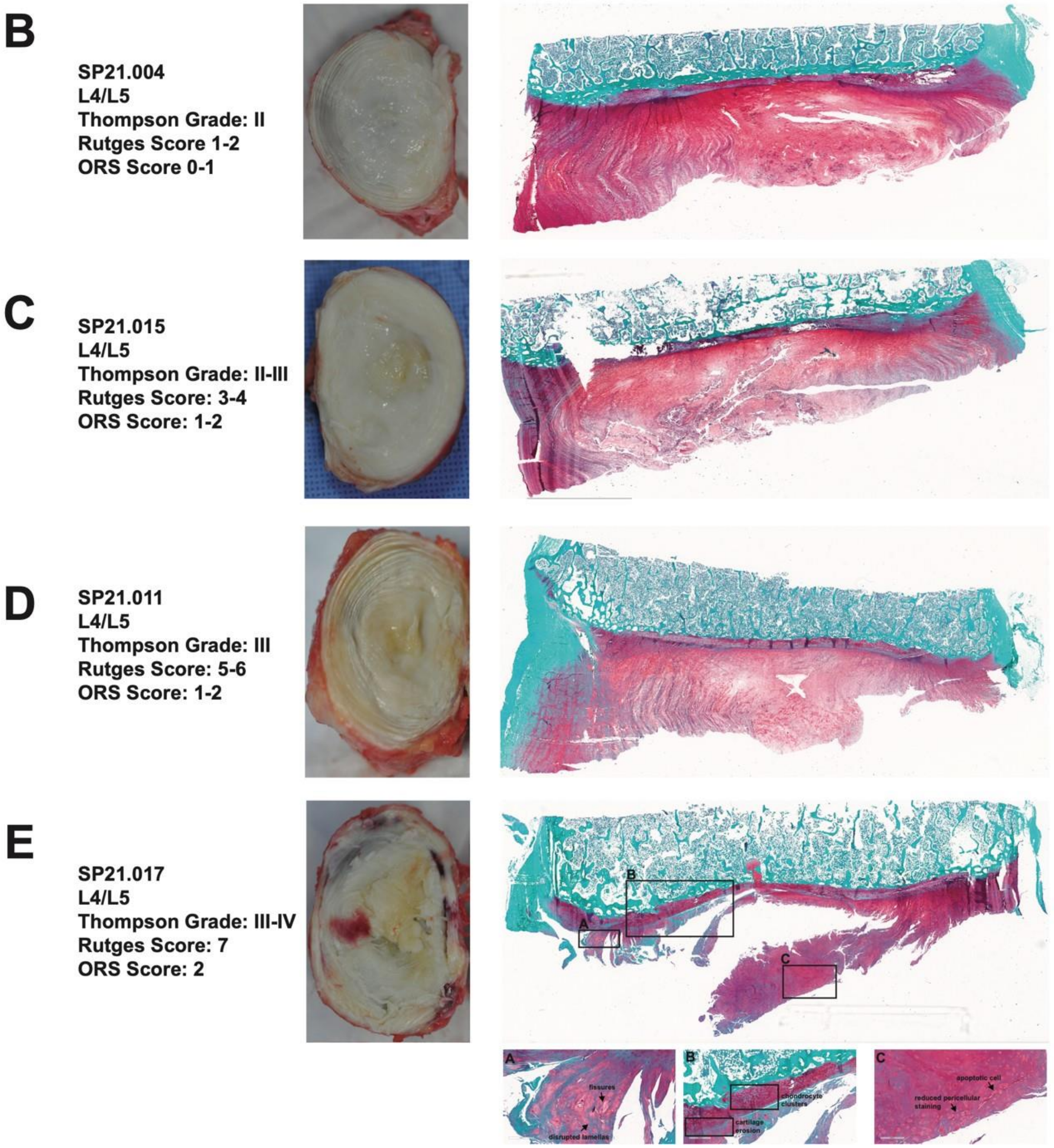

Figure S10. Classification of IVD grades. (A) Table showing representative donors and their Thompson grade, Rutges score and ORS score. Descriptions for each classification are included in the table. **(B-E)** Macroscopic images (left) and histology of representative donors with Thompson grades II **(B)**, II-III **(C)**, III **(D)** and III-IV **(E)**.

Table S1. Donor information.

Donor ID	Thompson Grade	Disease State	Age	Sex	Disc
SP21.015	II	healthy	21	M	T12/L1
SP21.018	II	healthy	27	M	L3/L4
SP22.001	II	healthy	25	M	T12/L1
SP21.007	II-III	diseased	43	M	L1/L2
SP21.014	II-III	diseased	37	M	L2/L3
SP21.011	III	diseased	42	M	L1/L2
SP21.013	III	diseased	63	M	T12/L1
SP21.016	III-IV	diseased	68	M	L4/L5
SP21.017	III-IV	diseased	61	M	L2/L3
SP22.002	III-IV	diseased	64	M	L2/L3
SP22.003	III-IV	diseased	63	M	L2/L3
SP20.002	III-IV	diseased	73	M	L1/L2
SP20.006	III-IV	diseased	56	M	L2/L3

Table S2. Cluster signatures of fibroblast and chondrocyte populations.

Population	Signature	References
**Fibroblasts**	VIM, PRG4, S100A4, ADIRF, FAP, ACTA2, NFKB, FSP1, Zeb2-NAT, FGF-2, FGF-18, FGF-8, FGFR1, FN1, HSP47, COL1A1, COL1A2	PMID: 23060229<br>PMID: 25749011<br>PMID: 35743056<br>PMID: 31614494
**Chondrocytes**	MGP, MMP3, MMP13, HSPA8, BPIFA2F, PTGES, SOX9, CEMIP, AKR1C, COMP, DENR, MELT, NCMAP, RUNX2, DLX5, C2ORF40, MT1F, HIF1A, PRELP, EPYC, CHI3L1, COL1A1, COL1A2, COL5A1, COL2A1, LOXL1, LUM, FBLN1,FBLN2, CD34, ACAN, MIA, CCNL1, KLF2, RSRP1	PMID: 34400611<br>PMID: 22686699<br>PMID: 11950957<br>PMID: 35409356<br>PMID: 30026257<br>PMID: 32616761<br>PMID: 34045450<br>PMID: 34200880
